# Emergency department-initiated interventions to reduce risk of recurrent falls in older adults: a systematic review and meta-analysis

**DOI:** 10.1093/ageing/afag227

**Published:** 2026-08-03

**Authors:** Wilmar Charmant, Sofie Jansen, Natasja M van Schoor, Ralph de Vries, Prabath Nanayakkara, Hanna C Willems, Nathalie van der Velde

**Affiliations:** Amsterdam UMC Locatie VUmc, Department of Internal Medicine, Section General and Acute Medicine, Amsterdam, the Netherlands; Amsterdam UMC Locatie VUmc, Amsterdam Public Health Research Institute, Quality of Care, Amsterdam, the Netherlands; Amsterdam UMC Locatie AMC, Department of Internal Medicine, Section Geriatric Medicine, Amsterdam, the Netherlands; Dijklander Hospital, Department of Geriatric Medicine, Hoorn, the Netherlands; Amsterdam UMC Locatie VUmc, Department of Epidemiology and Data Science, Amsterdam Public Health research institute, Amsterdam,the Netherlands; Amsterdam UMC Locatie VUmc, Amsterdam Public Health Research Institute, Ageing and Later Life, Amsterdam, the Netherlands; Vrije Universiteit Amsterdam, Medical Library, Amsterdam, the Netherlands; Amsterdam UMC Locatie VUmc, Department of Internal Medicine, Section General and Acute Medicine, Amsterdam, the Netherlands; Amsterdam UMC Locatie VUmc, Amsterdam Public Health Research Institute, Quality of Care, Amsterdam, the Netherlands; Amsterdam UMC Locatie AMC, Department of Internal Medicine, Section Geriatric Medicine, Amsterdam, the Netherlands; Amsterdam UMC Locatie VUmc, Amsterdam Public Health Research Institute, Ageing and Later Life, Amsterdam, the Netherlands; Amsterdam UMC Locatie AMC, Department of Internal Medicine, Section Geriatric Medicine, Amsterdam, the Netherlands; Amsterdam UMC Locatie VUmc, Amsterdam Public Health Research Institute, Ageing and Later Life, Amsterdam, the Netherlands

**Keywords:** falls, acute care, falls prevention, emergency department, multifactorial interventions, systematic review, older people

## Abstract

**Background:**

Older adults presenting to the emergency department (ED) following a fall are at high risk for recurrent falls, injuries and increased healthcare utilisation. This systematic review and meta-analysis evaluated the effectiveness of interventions initiated upon ED presentation in reducing falls (primary outcome) and fall-related outcomes (secondary outcomes) among older adults.

**Methods:**

A comprehensive search was performed in Ovid Medline, Embase, CINAHL, PEDro, Web of Science, and Scopus up to June 2025, following PRISMA guidelines. This yielded 9624 references, of which 4811 records remained after deduplication and were screened for eligibility. Meta-analyses and descriptive methods, including vote counting, were applied where appropriate. Sensitivity and subgroup analyses were conducted.

**Results:**

Thirty articles were included. Interventions were associated with a significant reduction in fall incidence (rate ratio 0.69; 95% CI 0.54–0.88; I^2^ = 95%), supported by vote counting. While reductions were also observed in the proportion of fallers (odds ratio 0.87; 95% CI 0.71–1.07; I^2^ = 61%), fall-related and all-cause ED revisits, hospital admissions, recurrent falls and mortality, these trends did not reach statistical significance. Sensitivity analysis excluding studies with modified usual care showed a significant reduction in fall-related ED revisits and people sustaining an injurious fall. Secondary outcomes including quality of life, fear of falling and physical functioning generally showed trends favouring the intervention group. Considerable heterogeneity and variation in intervention characteristics were noted across studies.

**Conclusion:**

Interventions for older adults presenting to the ED after a fall suggest a favourable effect on fall rate. Non-significant trends favouring the intervention group were observed for several other outcomes, but heterogeneity and methodological limitations preclude definitive conclusions.

## Key points

- Emergency department-initiated interventions for older adults presenting after a fall significantly reduce fall incidence.
- Non-significant trends favouring the intervention group were observed for fall-related ED revisits and hospital admissions.
- Non-significant trends favouring the intervention group were observed for quality of life and fear of falling.

## Introduction

Falls among older adults represent a growing healthcare burden, with substantial consequences at both individual and societal levels [1, 2]. Resulting injuries often require treatment, and loss of independence may lead to institutionalisation [3, 4]. Falls are the leading cause of emergency department (ED) visits and injury-related mortality in older adults, placing considerable strain on healthcare systems worldwide [5–8]. One in seven patients sustain a recurrent fall within 30 days of their index fall, and as fall frequency increases, the interval between subsequent falls tends to shorten [9]. For many patients, an ED visit following a fall injury is the only interaction with a healthcare professional regarding their fall, due to both patient- and system-related factors [10]. Falls are frequently and incorrectly regarded as an inevitable consequence of ageing, leading both patients and healthcare providers to assign a low priority to fall prevention [11]. Stigma, minimization of the event and lack of awareness that falls are preventable contribute to underreporting by patients [12]. Consequently, opportunistic case-finding in the ED and follow-up fall-prevention measures after fall-related ED visits should be prioritised at the point of care [2].

Traditionally, ED care for falls has focused primarily on treatment of injuries, often overlooking both acute and urgent underlying causes of the fall. This is unsurprising given the overcrowding and time constraints typical of ED settings [13]. However, substantial evidence indicates that future falls can be significantly reduced by identifying and addressing fall risk–increasing factors [14]. Patient motivation to engage in fall prevention is highest immediately after a fall, supporting recommendations that preventive interventions should be initiated at the point of care during the initial ED visit [15–17].

The aim of this systematic review was to investigate the effectiveness of interventions initiated in the ED for older adults presenting with falls, specifically in reducing subsequent falls, ED revisits and hospital readmissions compared with usual care. Secondary aims were to assess whether ED-initiated interventions are more effective than those using retrospective case-finding, whether multifactorial interventions are more effective than single or multiple interventions, and which patient groups benefit most from fall-prevention interventions.

## Methods

This systematic review was conducted in accordance with the Preferred Reporting Items for Systematic Reviews and Meta-Analyses (PRISMA) guidelines. The protocol was prospectively registered in the PROSPERO International Prospective Register of Systematic Reviews (CRD42024567682).

### Literature search

Systematic searches were conducted in Ovid Medline, Embase, CINAHL (Ebsco), PEDro, Web of Science (Core Collection) and Scopus from inception to 23 June 2025, in collaboration with a medical information specialist (RV). Search terms included (with synonyms and related terms): ‘Accidental falls’, ‘Fall prevention’, ‘Emergency department’, ‘Geriatric assessment’, ‘Aged’ and ‘Elderly’. Reference lists of included articles were screened for additional relevant publications. All languages were eligible. Duplicate references were removed using EndNote X21.0.1 (Clarivate™), following the Amsterdam Efficient Deduplication and Bramer methods [18, 19]. Full search strategies are provided in Supplement A.

### Selection criteria

Studies were included if they evaluated a fall-preventive intervention performed or initiated in the ED, including case-finding, in adults aged 60 years or older presenting to the ED due to a fall. Studies without an explicit age criterion were included if all participants were aged 60 years or older. The primary outcome was the number of falls during follow-up. Secondary outcomes included injurious falls, fallers, people sustaining an injurious fall, hip fractures, fall-related and all-cause ED revisits, fall-related and all-cause hospital admissions, quality of life, mortality, physical functioning, patient-reported outcome measures (PROMs), concerns about falling, participation level and other relevant outcomes.

Eligible study designs included randomised controlled trials (RCTs), non-randomised controlled trials (non-RCTs), interventional studies without concurrent controls, pre-post intervention studies, factorial designs and cluster-randomised studies. Studies involving non-ambulatory patients were excluded.

### Data extraction and quality assessment

Two reviewers (SJ and WMC) independently screened all titles and abstracts using ASReview. Full texts were reviewed when eligibility was unclear. Disagreements were resolved through consensus or consultation with a third reviewer (NV). Data extraction was performed on all included full-text articles.

Study quality was independently assessed by SJ and WMC using the appropriate National Heart, Lung, and Blood Institute Study Quality Assessment Tools [20]. Risk of bias was assessed using the Cochrane Risk of Bias 2 tool for RCTs [21] and ROBINS-I V2 for non-randomised and pre-post studies [22]. Disagreements were resolved through discussion, with adjudication by an additional reviewer (NvdV) when necessary.

### Data synthesis and meta-analysis

Study characteristics and participant demographics were summarised using appropriate descriptive statistics. Analyses followed the intention-to-treat principle. Meta-analyses using inverse variance–weighted random-effects models were conducted when three or more studies reported comparable outcomes. Effect measures included incidence rate ratios (iRRs), odds ratios (ORs) and hazard ratios (HRs) with 95% confidence intervals. Adjusted estimates were preferred when available. When necessary, effect estimates were calculated using Poisson regression in RStudio.

When heterogeneity exceeded 50% or outcome measures were too heterogeneous for meta-analysis, a descriptive synthesis was performed. Vote counting with a sign test based on direction of effect was used when five or more studies were available, irrespective of statistical significance [23]. Each study contributed one vote. A sign test evaluated whether the proportion of studies favouring the intervention differed significantly from 0.5. Outcomes reported by fewer than five studies were described narratively.

For outcomes reported at multiple time points, data from the longest follow-up were used. When multiple outcome measures represented the same construct, the most comparable measure was selected. Between-study heterogeneity was assessed using the I^2^ statistic, with values above 50% indicating substantial heterogeneity [24]. Sensitivity analyses explored robustness by excluding individual studies and examining differences in population characteristics and control group content. Analyses were performed using Review Manager version 5.2 (RevMan, The Cochrane Collaboration, Oxford, United Kingdom).

### Subgroup and sensitivity analyses

Predefined subgroup analyses were conducted by follow-up duration (1–6 vs. 7–12 months), age group (60–70 vs. ≥70 years), site of intervention delivery (ED vs. follow-up appointment), special populations (e.g. cognitive impairment or residential care) and intervention type. Intervention types were classified as single, multiple (standardised combination of interventions) or multifactorial (individualised combination of interventions) [25]. Sensitivity analyses excluded studies at high risk of bias, those with alternative control interventions and those including participants via non-ED acute care pathways.

## Results

### Search yield

**Figure 1:**
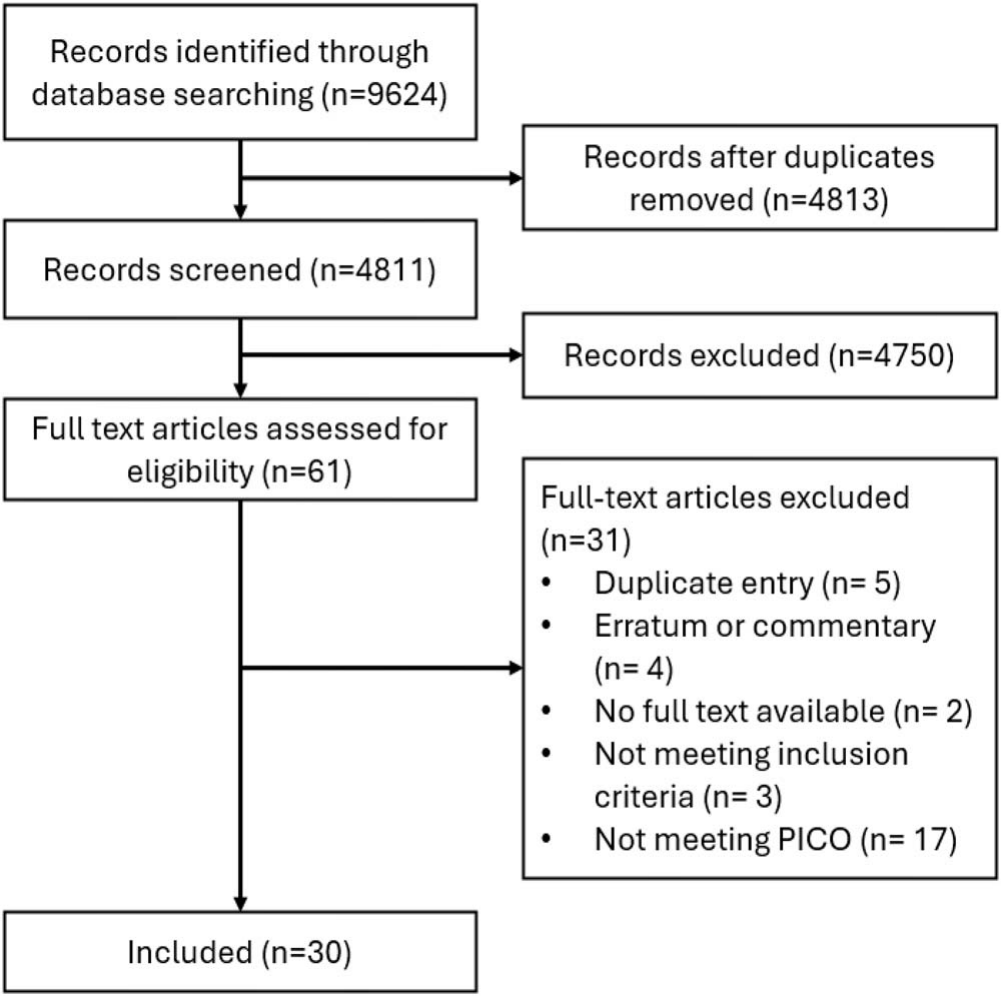
Flow chart.

The search identified 9624 references: 2150 from Ovid Medline, 3595 from Embase, 1470 from CINAHL, 34 from PEDro, 1190 from Web of Science and 1185 from Scopus. After removal of duplicates, 4811 references remained. The study selection process is illustrated in Figure 1.

### Study characteristics and outcomes

Thirty publications reporting on 27 unique studies were included [17, 26–54]. Of these, 17 were RCTs, 6 were non-RCTs and 4 used a pre-post design (Table 1). RCTs were published between 1999 and 2025, non-RCTs between 2013 and 2025 and pre-post studies between 1997 and 2024. Sample sizes ranged from 104 to 712 in RCTs, 41 to 2757 in non-RCTs and 217 to 5109 in pre-post studies. Studies were conducted in 11 countries, most commonly Australia (*n* = 6), the United Kingdom (*n* = 4) and the Netherlands (*n* = 4).

**Table 1:** Characteristics of included studies.

Author, year	Type of study	Country	Sample size	Inclusion criteria	Exclusion criteria	Mean age, % female (♀)	Follow-up	Outcome measures of interest
Barker 2018 [28]	RCT	Australia	441	60–90 years ED due to a fall Discharged Able to walk	Residential care, palliative care, terminal illness Non-English speaking, unable to use telephone Cognitive impairment Social aggression, history of psychosis	73y, ♀ 55%	12 months	No. of falls and injurious falls No. of ED revisits hospitalisations, fractures Quality of life ▪ Mortality
Benhamou 2025 [29]	RCT	Switzerland	104	65+ ED due to fall	Hospital admission, immobilising fractures Cognitively impaired Non-Swiss German speaking Inability to follow instructions	81y, ♀ 58%	42 days	Concerns about falling No. of fallers
Boyé 2017 [31], Polinder 2016 [48]	RCT	NL	612	65+ ED due to a fall Fall-risk increasing drugs Able to walk	Cognitive impairment Enrolled in another study Likely follow-up problems Not meeting fall definition	76y, ♀ 62%	12 months	Time to fall Fall-related ED revisit
Chu 2016 [32]	RCT	Hong Kong	204	65+ ED due to a fall Ambulatory	Fall due to alcohol, sudden blow or epileptic seizure Cognitive impairment Non-Cantonese speaking Nursing home	78y, ♀ 71%	12 months	No. of falls and fallers No. of recurrent fallers Time to next fall-related ED revisits Physical functioning
Close 1999 [34]	RCT	UK	397	65+ ED due to a fall	Cognitive impairment Out of the area Non-English speaking	78y, ♀ 68%	12 months	No. of falls and injurious falls Mortality, physical functioning All-cause hospital admission
Dadgari 2022 [35]	RCT	Iran	112	60+ Short hospitalisation due to fall Living with a family member	Similar training during previous admission Acute physical illness Psychotropic medication Transfer to another hospital, immobility	76y, ♀ 53%	6 months	No. of recurrent falls All-cause hospital admissions Injurious falls Physical functioning
Davison 2005 [36]	RCT	UK	313	65+ ED due to a fall Fall in preceding year	Cognitive impairment Previous syncope Immobile, blind, aphasic Medical cause of fall Enrolled in another study, out of the area	77y, ♀ 72%	12 months	No. of falls and fallers Injury rates Fall-related hospitalisation Mortality Physical functioning
Goldberg 2020 [39]	RCT	USA	110	65+ ED due to a fall English or Spanish speaking Discharged	Altered mental status Homeless, no follow-up possible Residing in skilled nursing facility	81y, ♀ 67%	6 months	No. of fall-related ED revisits No. of all-cause ED revisits No. of fall-related hospitalisations No. of all-cause hospitalisations
Harper 2017 [40]	RCT	Australia	123	65+ ED due to a fall Assessment by care team Residing in area Safe for discharge home	Cognitively impaired Non-English speaking Residing in nursing home Fall clinic in the past 6 months Enrolled in another intervention	82y, ♀ 66%	35 days	No. of falls Time to next fall
Hendriks 2008 [42]	RCT	NL	333	65+ ED or acute GP services due to a fall	Non-Dutch speaking Cognitive impairment Admitted >4 weeks Permanently wheelchair-dependent or non-ambulatory	75y, ♀ 70%	12 months	No. of falls and recurrent falls No. of injurious falls Time to next fall Physical functioning Concerns about falling Quality of life
Lightbody 2002 [45]	RCT	UK	348	65+ ED due to a fall	Admitted to hospital Living in institutional care Refusing consent Out of the area	75y, ♀ 74%	6 months	No. of falls and fallers Barthel index, life space diameter No. of ED revisits, fall-related GP visits, fall-related hospital admissions
Matchar 2017 [46], Matchar 2019 [47]	RCT	Singapore	354	65+ ED due to a fall Unimpaired cognition Discharged home/short admission	Traffic collision Emergency surgery	78y, ♀ 78%	9 months	No. of fallers No. of injurious falls Physical functioning
Russell 2010 [50]	RCT	Australia	712	60+ ED due to a fall Discharged home	Cognitive impairment Inability to follow instructions Inability to walk independently	75y, ♀ 70%	12 months	No. of falls No. of injuries, fractures Fall-related and all-cause ED revisits Fall-related and all-cause hospital days
Shaw 2003 [51]	RCT	UK	308	65+ ED due to a fall Cognitive impairment	Unable to walk, medical cause of fall Unfit for investigation Unable to communicate Out of the area No major informant available	84y, ♀ 80%	12 months	No. of falls and fallers Time to fall, injury rates Fall-related ED attendance Fall-related hospital admissions Mortality
Vind 2009 [53], Vind 2010 [52]	RCT	Denmark	392	65+ ED due to a fall	Fall due to external cause or alcohol Out of the area, institutionalised Immobile, terminally ill, impaired communication, dementia Planned geriatric intervention	74y, ♀ 74%	12 months	No. of falls and injurious falls No. of fallers and recurrent fallers Time to first fall and first injury
de Vries 2010 [37]	RCT	NL	217	65+ ED or GP practice due to a fall Living independently Assisted living facility Residing in area	Low risk of recurrent falls Inability to sign informed consent, cognitive impairment Fall due to traffic or occupational accident Residing in nursing home or long-term rehabilitation facility Fall >3 months before randomization	80y, ♀ 71%	12 months	Time to fall ADL Quality of life Physical performance
Whitehead 2003 [54]	RCT	Australia	140	65+ ED due to a fall	Nursing home Cognitive impairment	78y, ♀ 71%	6 months	No. of falls
Bogucki 2023 [30]	non-RCT	USA	2757	ED due to a fall No age cut-off	Long term care facility	76y, ♀ 67%	3 months	No. of all-cause ED revisits
Clementz 2019 [33]	non-RCT	France	252	75+ ED due to a fall ≥2 chronic diseases and ≥2 medications	Resuscitation Palliative care	NR	3 months	No. of all-cause hospital admissions
Fulbrook 2025 [38]	non-RCT	Australia	630	70+ ED due to a fall	Hospital admission at visit	83y, ♀ 57%	3 months	No. of fall-related ED revisits No. of all-cause ED revisits
Harper 2013 [41]	non-RCT	Australia	3346	65+ ED due to a fall	NR	83y, ♀ 70%	30 days	No. of ED revisits No. of hospital admissions
Hepkema 2022 [17]	non-RCT	NL	41	70+ ED due to a fall Living (semi) independent	Non-Dutch speaking Hospital admission Discharge to residential care facility Enrolled in another fall prevention study High impact fall	82y, ♀ 59%	3 months	No. of recurrent falls
Leahy 2014 [44]	non-RCT	Australia	115	ED due to a fall Medically stable Possible benefit from the intervention	NR	84y, ♀ 66%	6 months	No. of fall-related ED revisits
Ageron 2016 [26]	pre-post Δ	France	5109	75+ ED due to a fall	Out of the area Hospital admission Nursing home Dementia	85y, ♀ 71%	1 month	No. of fall-related ED revisits Hospitalisation Length of stay in hospital
Baraff 1999 [27]	pre-post Δ	USA	1899	65+ ED due to a fall	Fall due to blow, syncope, sport, work or acute medical event Hospital admission, living in nursing home, terminal illness, unable to walk or communicate Non-English or non-Spanish speaking Enrolled in another study, out of the area, not a member of the care organisation	76y, ♀ 73%	12 months	No. of falls Physical functioning Health care utilisation
Jusmanova 2021 [43] Ω	pre-post Δ	Ireland	1428	ED due to a fall	Fall due to sport, intoxication, drug overdose or alleged assault	77y, ♀ 61%	3 months	No. of hospital admissions Length of stay No. of hospital readmissions
Rosario 2023 [49]	pre-post Δ	Singapore	217	ED due to a fall	NR	79y, ♀ 56%	6 months	No. of ED revisits No. of hospital admissions Mortality

ADL: activities of daily living; Δ pre-post: pre-post intervention study, Nr: number, NR: not reported, NL: the Netherlands, USA: United States of America, UK: United Kingdom, GP = general practitioner. Ω This study had no specific age requirement for inclusion; from this study results were obtained specifically of those patients aged 60 years and over to allow inclusion of their data by the original authors.

Participants were predominantly community-dwelling older adults presenting to the ED after a fall. Most studies used a lower age threshold of 65 years, although some included participants aged 60 years or older. Specific populations included patients with cognitive impairment [51], those with multiple chronic conditions and polypharmacy [33] and a small proportion recruited via general practitioner practices [37]. Mean participant age ranged from 73 to 85 years, with women comprising 53% to 80% of samples. Interventions were classified as multifactorial (*n* = 22), multiple (*n* = 1) or single (*n* = 4). Follow-up durations ranged from 30 days to 12 months, most commonly 6 or 12 months.

### Care as usual

Most studies compared interventions with care as usual, defined as no study-driven intervention. However, three RCTs deviated slightly from this definition. Chu et al. conducted a non-interventional ‘well-wishing’ home visit in the control group [32]. Shaw et al. performed a multifactorial assessment in all participants prior to randomization, without subsequent intervention in controls [51]. Russell et al. informed control participants of their fall risk and advised consultation with their general practitioner [50].

### Methodological quality and risk of bias of included studies

Quality of included RCTs was variable, with scores on the NIH Quality Assessment Tool for Controlled Intervention Studies ranging from 5 to 13 (Table 2). The most frequently observed methodological concerns in the RCTs were a lack of blinding of participants, providers and outcome assessors, high drop-out rates, low adherence and insufficient sample size for at least 80% power (Supplement B). The non-RCTs were also assessed with the NIH tool for Controlled Intervention Studies, with scores ranging from 1 to 9. The pre-post intervention studies were evaluated using the NIH Quality Assessment Tool for Pre-Post Studies Without a Control Group, with total scores ranging from 2 to 9.

**Table 2:** Intervention description, quality assessment and risk of bias assessment of the included studies.

Author, year	Type of intervention	Intervention group	Control group	Quality and risk of bias
RCTs				
Barker 2018 [28]	Multifactorial	FROP-Com assessment, home safety assessment, identification of fall risk factors, education leaflet with four modules (better strength, better vision, better sleep, better bones). Encouragement to choose one or more modules through motivational interviewing.	Care as usual	NIH_CT_: **10** RoB2: Low risk
Benhamou 2025 [29]	Single Δ	Physiotherapeutic assessment, education, exercise prescription, self-management instructions and fall prevention information in the ED	Care as usual	NIH_CT_: **5** RoB2: High risk
Boyé 2017 [31], Polinder 2016 [48]	Single	Geriatric outpatient clinic assessment including fall-related medication assessment and safely adjusted if possible.	Geriatric outpatient clinic, without specific fall-related medication withdrawal (care as usual)	NIH_CT_: **11** RoB2: Low risk
Chu 2016 [32]	Single	Environmental hazard evaluation (1.5-h) by OT with potential home modifications. Follow-up call after 2 months. Second home visit if necessary.	Single ‘well-wishing’ visit by a research assistant (1.5 hours)	NIH_CT_: **11** RoB2: High risk
Close 1999 [34]	Multifactorial	Comprehensive assessment of visual acuity, balance, cognition, medication, postural hypotension. Carotid sinus studies if cause of fall unclear or high clinical suspicion. Referrals to day hospital. Drug modification by direct contact with the GP. A single home visit by OT.	Care as usual	NIH_CT_: **9** RoB2: High risk
Dadgari 2022 [35]	Multiple	Four predefined sessions and a home visit consisting of education on falls and fall risk factors, medication assessment, home safety assessment, training of motor skills.	Care as usual	NIH_CT_: **10** RoB2: High risk
Davison 2005 [36]	Multifactorial	Hospital-based assessment (medical and fall history, clinical examination, medication, vision, orthostatic hypotension, carotid sinus hypersensitivity and vasovagal hypersensitivity). Laboratory tests and ECG. Gait and balance, feet, footwear and assistive devices, home-based PT and OT assessment. Interventions for identified risk factors.	Care as usual	NIH_CT_: **13** RoB2: High risk
Goldberg 2020 [39]	Multifactorial Δ	Medication review with identification of 1–3 medications that could be stopped or modified to reduce fall risk. Bedside PT evaluation and assessment of gait, balance and lower extremity strength.	Care as usual	NIH_CT_: **10** RoB2: High risk
Harper 2017 [40]	Multifactorial	Fall risk screening at the ED, OT home visit with individualised assessment and targeted interventions, weekly/fortnightly phone calls until falls specific outpatient service.	Falls specific outpatient service (care as usual)	NIH_CT_: **8** RoB2: High risk
Hendriks 2008 [42]	Multifactorial	Medical and occupational-therapy assessment to evaluate and address risk factors for recurrent falls, followed by recommendations and referral if indicated.	Care as usual	NIH_CT_: **11** RoB2: High risk
Lightbody 2002 [45]	Multifactorial	Home fall risk assessment by nurse specialist, including medication, home safety, ECG, blood pressure, cognition, visual acuity, hearing, vestibular dysfunction, balance, mobility, feet and footwear. Referrals to relatives, community therapy services, social services or primary care team. No direct referrals to outpatient clinics.	Not specified	NIH_CT_: **6** RoB2: High risk
Matchar 2017 [46], Matchar 2019 [47]	Multifactorial	Medical examination, tailored program of PT focused on strength, balance and gait for a period of 3 months, as well as screening and follow-up for vision, polypharmacy and environmental hazards. Plus educational material.	Care as usual	NIH_CT_: **13** RoB2: Some concerns
Russell 2010 [50]	Multifactorial	FROP-Com assessment. Targeted referrals to PT, OT, podiatry, dietetics and family physician. Health promotion recommendations including advice to make an appointment with an optometrist, purchase hip protectors, improve footwear safety and make minor home improvements.	Standard care arranged by ED. Letter to patient informing of FROP-Com falls risk and recommendation to speak to family physician about fall risk.	NIH_CT_: **12** RoB2: High risk
Shaw 2003 [51]	Multifactorial	Multifactorial clinical assessment (medical, PT, OT and cardiovascular) at baseline with interventions for all identified risk factors.	Same assessment without interventions for identified risk factors.	NIH_CT_: **13** RoB2: Some concerns
Vind 2009 [53], Vind 2010 [52]	Multifactorial	Assessment of medical, cardiovascular and physical ability by doctor, nurse, PT in two visits. Interventions for identified risk factors.	Care as usual	NIH_CT_: **12** RoB2: Low risk
de Vries 2010 [37]	Multifactorial	Assessment of fall risk factors followed by subsequent interventions, including medication withdrawal, treatment of OH, PT, OT, referrals to other medical specialists if indicated.	Care as usual	NIH_CT_: **10** RoB2: Some concerns
Whitehead 2003 [54]	Multifactorial	Fall risk assessment, followed by GP initiated interventions like medication review, home assessment by OT, exercise program, falls clinic or assessment of osteoporosis.	Care as usual	NIH_CT_: **11** RoB2: Some concerns
Non-RCTs				
Bogucki 2023 [30]	Multifactorial	Home safety check, medication reconciliation, assessment of frailty, determination of needs for physical or OT. Interventions for identified risk factors.	Care as usual	NIH_CT_: **3** ROBINS-I: Serious risk
Clementz 2019 [33]	Single	In-hospital medication reconciliation, highlighting discrepancies followed by medication review.	Care as usual	NIH_CT_: **6** ROBINS-I: Serious risk
Fulbrook 2025 [38]	Multifactorial	Multifactorial falls risk screening tool with referrals to physiotherapy, pharmacy, podiatry, drug and alcohol and ambulatory care team.	Care as usual	NIH_CT_: **9** ROBINS-I: Serious risk
Harper 2013 [41]	Multifactorial Δ	ED Care Coordination Team can initiate fall risk assessment, education, functional retraining, supply of equipment and referrals to falls clinics or outpatient allied health services.	Care as usual	NIH_CT_: **7** ROBINS-I: Critical risk
Hepkema 2022 [17]	Multifactorial	Multifactorial fall risk screening followed by personalised interventions.	Care as usual	NIH_CT_: **2** ROBINS-I: Low risk ∞
Leahy 2014 [44]	Multifactorial	10-week multi-disciplinary program of education and exercise for fall patients. One-to-one sessions with relevant health staff, medical review by a geriatrician, fall-specific education and exercise by PT and OT, and access to other allied health services (dietician, clinical psychologist and podiatrist) as required.	Care as usual	NIH_CT_: **1** ROBINS-I: Serious risk
Pre-post intervention studies				
Ageron 2016 [26]	Multifactorial Δ	Short assessment of risk factors and recommendation for blood test and ECG. Triage after ED discharge with four options: return home with advice, return home with a subsequent visit to a geriatrician for a complete assessment, intervention of a geriatric team in the ED before discharge at home and hospitalisation with an alert to the geriatric team	Care as usual	NIH_PP_: **9** ROBINS-I: Low risk
Baraff 1999 [27]	Multifactorial Δ	Geriatric assessment and if appropriate: medication reconciliation, alcohol abuse intervention, social service referral, home health referral, PT referral, optometry referral, podiatry referral, exercise, immunizations, calcium and vitamin D supplements.	Care as usual	NIH_PP_: **6** ROBINS-I: Critical risk
Jusmanova 2021 [43]	Multifactorial Δ	Initial ED assessment with autonomic function testing (e.g. tilt-table, finometer, ambulatory monitoring). Rapid access to multidisciplinary team and day hospital. Management based on guidelines.	Care as usual	NIH_PP_: **7** ROBINS-I: Low risk
Rosario 2023 [49]	Multifactorial Δ	Comprehensive geriatric assessment in the ED	Care as usual	NIH_PP_: **2** ROBINS-I: Critical risk

♀ female. ∞ Not all domains could be assessed. PT: physical therapy or therapist. OT: occupational therapy or therapist. NIH_CT_: National Heart, Lung, and Blood Institute Study Quality Assessment Tool for Controlled Intervention Studies. NIH_PP_: National Heart, Lung, and Blood Institute Study Quality Assessment Tool for Before-After (Pre-Post). RoB2: Risk of Bias Tool for randomised trials Cochrane. ROBINS-I: Risk of Bias in Non-Randomised Studies – of Interventions Cochrane, GP = general practitioner. Quality assessment: NIH_CT_ (max 14 points), NIH_PP_ (max 12 points). Risk of bias categories: low risk, some concerns, high risk of bias. ROBINS-I: low risk, moderate risk, serious risk, critical risk of bias. Δ Intervention partly or completely performed in ED, excluding fall risk assessments-only.

Using the RoB 2 tool, 10 RCTs were judged to be at high risk of bias, 4 raised some concerns and 3 were judged to have a low risk of bias. Concerns regarding bias most frequently occurred from assignment and adherence to intervention (Supplement C). Based on the ROBINS-I tool, one non-RCT was judged to be at critical risk of bias, four at serious risk and one at low risk. According to ROBINS-I, two of pre-post designs were judged to have a critical risk of bias and two a low risk. Outcomes per study are reported in Supplement D.

## Effectiveness of interventions

### Incidence (rate) of falls

**Figure 2:**
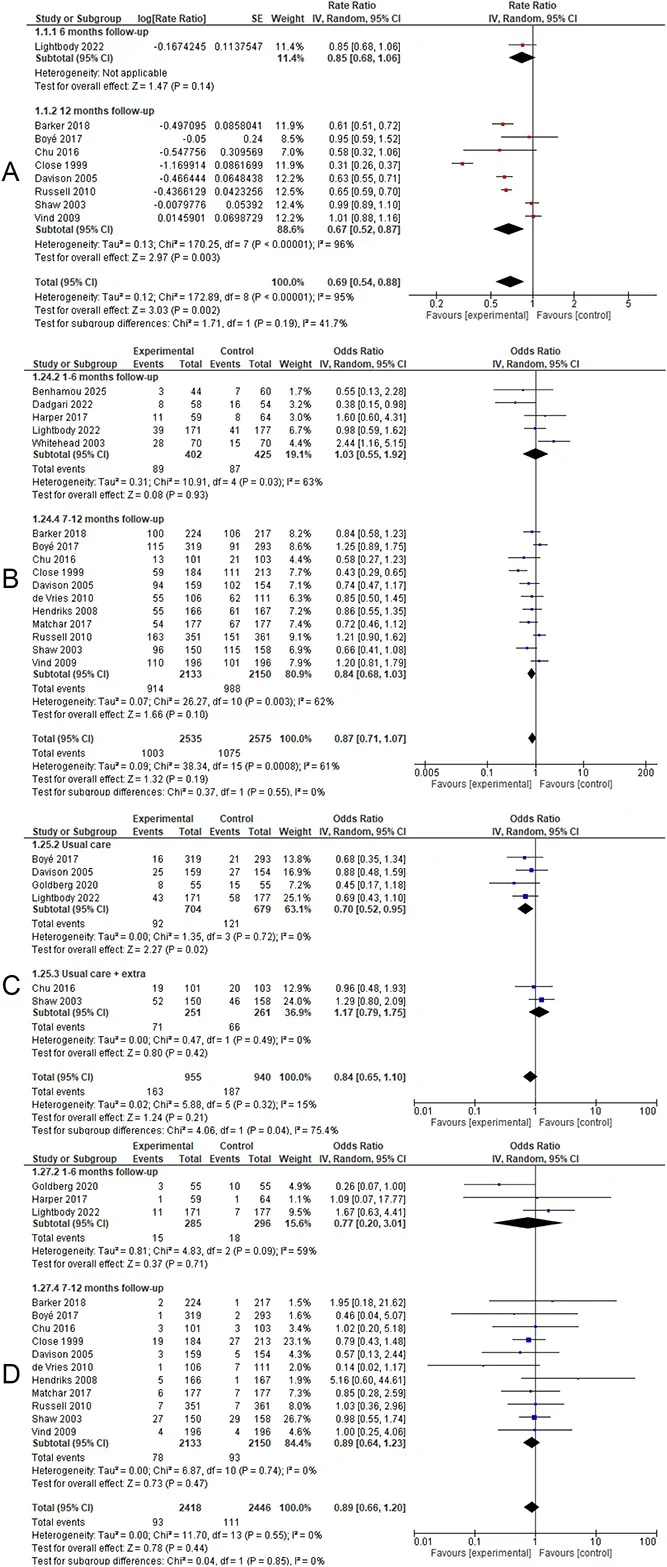
Meta-analysis of effect of fall prevention programs on fall outcomes: (A) falls, (B) proportion of fallers, (C) fall-related ED revisits and (D) mortality.

Meta-analysis of 9 RCTs including 3727 participants demonstrated a statistically significant reduction in fall incidence in the intervention group compared with usual care (iRR 0.69, 95% CI 0.54–0.88; I^2^ = 95%) [28, 31, 32, 34, 36, 45, 50, 51, 53] (Figure 2). Substantial heterogeneity indicated marked variability across studies. Vote counting showed that eight of nine studies favoured the intervention (88%, 95% CI 51.8%–99.7%, *P* = .04). No non-RCTs reported fall incidence, and one pre-post study found no difference (*P* = .993) [27].

### Fallers

Sixteen RCTs involving 5110 participants showed a non-significant reduction in the proportion of patients experiencing a fall after intervention (OR 0.87, 95% CI 0.71 to 1.07; I^2^ = 61%) [28, 29, 31, 32, 34–37, 40, 42, 45, 46, 50, 51, 53, 54] (Figure 2). Vote counting favoured the intervention in 11 of 16 studies (69%, 95% CI 41%–89%; *P* = .21). One non-RCT reported fewer fallers in the intervention group (20% vs. 32%) [17]. While one pre-post study found similar proportions pre- and post-intervention (21.1% vs. 18.2%) [27].

### People sustaining an injurious fall

Meta-analysis of five RCTs (*n* = 2188) showed a non-significant reduction in people sustaining an injurious fall in the intervention group (OR 0.82, 95% CI 0.61–1.10, I^2^ = 32%) [34, 42, 46, 50, 53]. One pre-post study found no difference in people sustaining an injurious fall requiring hospitalisation (3.2% vs. 2.9%; *P* = .74) [27].

### Recurrent fallers

Seven RCTs (*n* = 2596) showed a non-significant reduction in the odds of experiencing two or more falls during follow-up [28, 31, 32, 34, 37, 42, 53]. Vote counting favoured the intervention in four studies (57.1%, 95% CI 28.4%–99.5%, *P* = .99). One pre-post study also reported no significant difference (8.0% vs. 9.7%, *P* = .25) [27].

### Hip fractures

Pooled data from four RCTs (*n* = 1454) showed a non-significant reduction in hip fractures (OR 0.52, 95% CI 0.26–1.02; I^2^ = 0%) [28, 36, 51, 53]. A pre-post study similarly found no difference (1.3% vs. 1.1%; *P* = .60) [27].

### Time to first fall

Four RCTs including 1554 participants found no difference in time to first fall after ED discharge (HR 1.09, 95% CI 0.94–1.27; *P* = 0.25, I^2^ = 0%) [31, 37, 42, 53].

### Fall-related ED revisits

Three RCTs (*n* = 1214) examining fall-related ED revisit rates found a non-significant reduction (iRR 0.84, 95% CI 0.45 to 1.58; I^2^ = 77%), with substantial variation between studies (*Goldberg et al., 2020:* 0.34, 95% CI 0.15–0.76; *Russell et al., 2010:* 1.03, 95% CI 0.68–1.54; *Vind et al., 2009*: 1.26, 95% CI 0.79–2.01) [39, 50, 53].

Six RCTs (*n* = 1895) assessing the proportion of patients with fall-related ED revisits also showed a non-significant reduction (OR 0.84, 95% CI 0.65 to 1.10; I^2^ = 15%) [31, 32, 36, 39, 45, 51] (Figure 2). Two non-RCTs reported reductions with ORs of 0.36 (95% CI 0.16 to 0.80) and 0.99 (95% CI 0.54 to 1.81) [38, 44]. One pre-post study showed a reduction after covariate adjustment (OR 0.52, 95% CI 0.28 to 0.95) [26].

### All-cause ED revisits

Three RCTs (*n* = 1263) found a non-significant reduction in all-cause ED revisit rates (iRR 0.83, 95% CI 0.56–1.23, I^2^ = 85%) [28, 39, 50]. One RCT reported a non-significant reduction in proportions (OR 0.51, 95% CI 0.24–1.10) [39]. Two non-RCTs showed favourable effects with ORs of 0.62 (95% CI 0.53–0.72) and 0.97 (95% CI 0.78–1.22) [30, 38], while one reported a non-significant increase (OR 1.31, 95% CI 0.96–1.80) [41]. One pre-post study found no difference within 30 days (*P* = .831) [49].

### Fall-related hospital admissions

Results for the incidence of fall-related hospital admissions varied across studies. One RCT found an increase in incidence (iRR 2.33, 95% CI 1.30–4.18), while another found no difference (iRR 0.99, 95% CI 0.31–3.27) [39, 53]. One other RCT found no difference in fall-related days in the hospital (iRR 2.33, 95% CI 0.71–7.67) [50]. One pre-post study found a non-significant reduction with an iRR of 0.94 (95% CI 0.58–1.52) [27]. Meta-analysis of five RCTs (*n* = 1283) showed no significant difference in the proportion of fall-related admissions (OR 0.88, 95% CI 0.59–1.31; I^2^ = 0%) [34, 42, 46, 50, 53]. One pre-post study also found no difference (3.0% vs. 3.4%; *P* = .64) [27].

### All-cause hospital admissions

Two RCTs found non-significant reductions in all-cause hospital admission with iRR of 0.57 (95% CI 0.31–1.04) and 0.78 (95% CI 0.55–1.10) [28, 39]. One study found a significant mean reduction in all-cause hospitalisations (mean difference −0.24; 95% CI −0.40 to −0.08) [35]. Another RCT found no difference in hospital days (iRR 1.29, 95% CI 0.75–2.22) [50].

Regarding proportions, two RCTs showed non-significant reductions with ORs of 0.61 (95% CI 0.35–1.05) and 0.50 (95% CI 0.22–1.15) [34, 39]. In contrast, one non-RCT found an increase in proportion with an OR of 1.41 (95% CI 0.97–2.04), while another found a reduction (OR 0.45, 95% CI 0.26–0.79) [33, 41]. Pooled data from three pre-post studies (*n* = 3544) demonstrated a significant reduction in all-cause hospital admissions (OR 0.81, 95% CI 0.68–0.98; *P* = .03, I^2^ = 11%) [27, 43, 49].

### Mortality

Fourteen RCTs (*n* = 4860) showed a non-significant reduction in mortality (OR 0.89, 95% CI 0.66–1.20, I^2^ = 0%) [28, 31, 32, 34, 36, 37, 39, 40, 42, 45, 46, 50, 53] (Figure 2). One non-RCT and one pre-post study also found no significant differences [33, 49].

### Quality of life and participation

Six RCTs assessed quality of life, with four favouring the intervention (vote counting 66%, 95% CI 22%–96%, *P* = .69) [28, 37, 42, 46, 48, 52]. Three RCTs reported mixed effects on participation outcomes, including improved ability to go out alone (77% vs. 65%, *P* = .04) [34], fewer problems with usual activities [28] and no effect on social participation [42].

### Concerns about falling and functioning

Five RCTs evaluated concerns about falling (fear of falling), with three favouring the intervention (vote counting: 60%, 95% CI 14.7%–94.7%, *P* = .99) [28, 29, 36, 42, 52]. Eleven RCTs assessed physical functioning, with seven favouring the intervention (vote counting: 63.6%, 95% CI 30.8%–89.0%, *P* = .55) [28, 32, 34, 35, 37, 40, 42, 45, 46, 48, 52]. Outcome heterogeneity precluded meta-analysis.

### Subgroup and sensitivity analyses

A positive but non-significant trend for reduced fallers and mortality was observed in studies with longer follow-up (6–12 months) (Figure 2). Subgroup analyses for setting of intervention, type of intervention, age groups or specific patient populations were limited by small numbers.

Excluding 10 RCTs with high risk of bias resulted in attenuated effects across outcomes: 4 studies showed an iRR of 0.87 (95% CI 0.67–1.12; I^2^ = 89%) for number of falls; 6 studies an OR of 0.99 (95% CI 0.77–1.29; I^2^ = 57%) for fallers; 7 studies an OR of 0.84 (95% CI 0.55–1.30; I^2^ = 0%) for mortality; 4 studies an OR of 0.99 (95% CI 0.77–1.26; I^2^ = 6%) for recurrent fallers; and 3 studies an HR of 1.10 (95% CI 0.92–1.30; I^2^ = 0%) for time to first fall. Too few RCTs remained for meta-analysis of other outcomes.

Excluding three studies with modified control care showed significant reductions in fall-related ED revisits (OR 0.70, 95% CI 0.52–0.95; I^2^ = 0%) and people sustaining an injurious fall (OR 0.71, 95% CI 0.52–0.97; I^2^ = 0%) [32, 50, 51]. No changes were observed for other outcomes. Excluding one study, with 15% recruitment via general practitioner practices, did not alter point estimates [37]. Sensitivity analyses for non-RCT and pre-post studies were not informative due to limited numbers.

## Discussion

This systematic review indicates that fall-prevention interventions initiated in the ED are effective in reducing fall incidence among older adults. Other outcomes showed positive but non-significant trends, including fallers, fall-related and all-cause ED revisits, mortality, recurrent falls, hip fractures, hospital admissions, injurious falls, quality of life, fear of falling, physical functioning and participation. However, substantial heterogeneity and variability in intervention components limit the strength and generalisability of these findings. Moreover, too few studies were conducted exclusively in the ED or in specific patient populations to allow meaningful subgroup comparisons.

### Comparison with previous studies

Previous systematic reviews have examined multifactorial fall-prevention interventions following fall-related ED visits [15, 16]. With the publication of new studies and additional outcome data, an updated review was warranted. Overall, our findings align with earlier reviews. Harper et al. (2021) reported a significant reduction in fall incidence (iRR 0.69, 95% CI 0.52–0.91) but not in the proportion of fallers (RR 0.93, 95% CI 0.82–1.06) [15]. Similarly, our review found a significant reduction in fall incidence (iRR 0.69, 95% CI 0.54–0.88; I^2^ = 95%), with other outcomes showing non-significant favourable trends. Morello et al. (2019) included 12 RCTs and found no statistically significant effects, although effect estimates were close to significance and in a comparable direction (e.g. iRR 0.78, 95% CI 0.58–1.05) [16]. Reviews of community-based interventions reported similar reductions in fall incidence [14, 55], with stronger effects observed in high-risk populations [56].

Several factors may explain the generally positive but non-significant trends. Comparisons were made with usual care rather than no care, meaning control participants retained access to healthcare and may have been more aware of fall risk. Participation in a fall-prevention study itself may have prompted behavioural changes. Control participants in some trials received substantial fall-related care, such as referrals to specialists or physical therapy [37]. To illustrate, in one trial, 48% of the control group actively engaged in preventive strategies [54]. Additionally, informing general practitioners about fall risk may have triggered care outside the intervention, particularly given the widespread availability of fall-prevention services in Western countries.

Falls are inherently multifactorial, and the number and complexity of risk factors vary across individuals, as do responses to interventions, complicating the evaluation of interventions. Methodological challenges further affected observed effects, including inability to blind participants and providers, high dropout rates reflecting population frailty and low adherence to interventions (Supplements B and C). Low adherence in intervention groups combined with increased prevention efforts in control groups likely diluted true effects. Given the consistent direction of effects, improved adherence might have yielded statistically significant results. Future research should prioritise strategies to enhance engagement, ensure adequate sample sizes and blind outcome assessors where possible.

Older adults presenting to the ED after a fall are at high risk of recurrent falls, as previous falls are among the strongest predictors of future events [2, 57]. The ED visit may represent a critical ‘teachable moment’, during which patients are particularly receptive to preventive strategies [17, 58]. Initiating fall-prevention interventions at this point may therefore be especially effective, particularly given that fall-prevention management in community-dwelling older adults remains poor [2, 59–61].

**Figure 3:**
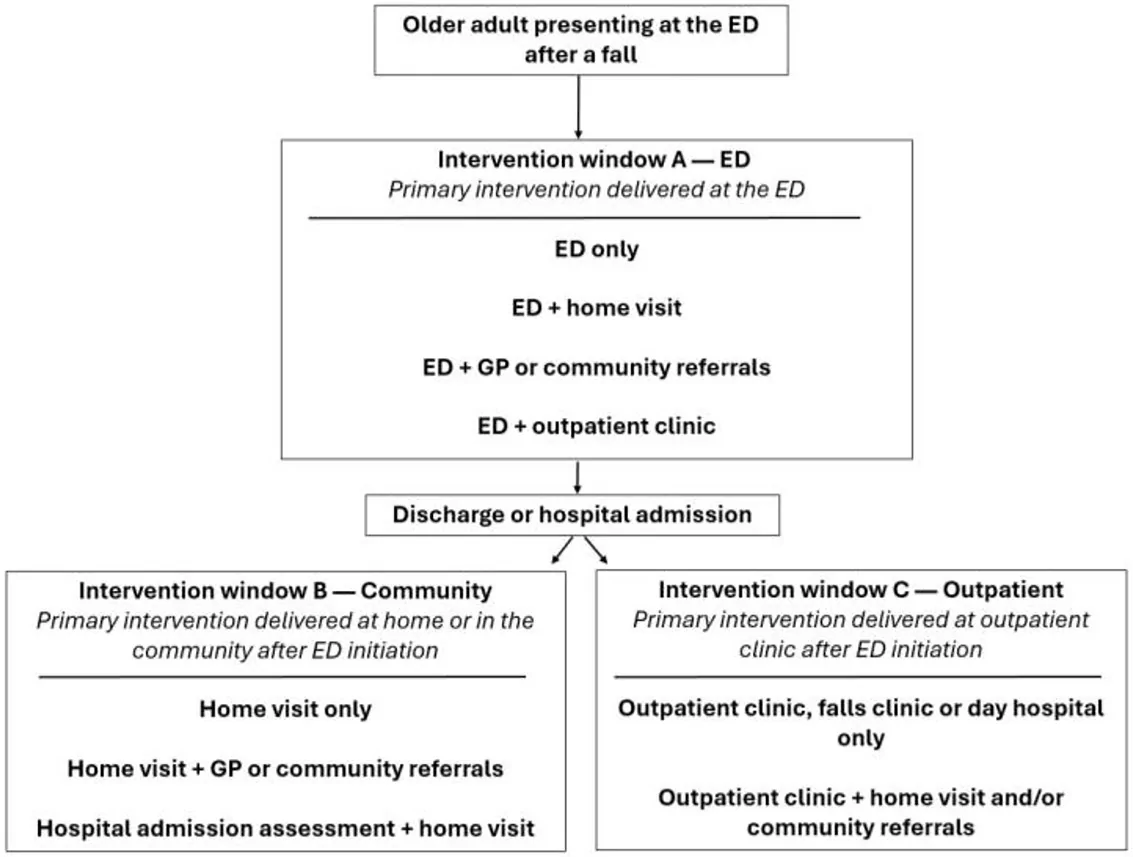
Visual summary of intervention windows for fall preventive care using the ED as entry point for case-finding. ED: emergency department; GP: general practitioner.

Various models of fall-preventive interventions using the ED as an entry point for case-finding were derived from this review, as shown in Figure 3. These included not only ED-initiated interventions, but also different trajectories of care following the ED visit, such as referral-based models involving primary care or community services, home-based follow-up and outpatient or falls clinic pathways, often in combination. This suggests that fall prevention after an ED visit can be organised through multiple care pathways and adapted to local context, available resources and existing care structures.

Within the ED itself, the studies in this review suggest several opportunities to initiate fall prevention already during the acute care encounter, including comprehensive geriatric assessment or brief fall risk factor assessment, medication review, physiotherapy assessment and targeted diagnostic evaluation such as blood tests or electrocardiography when relevant. Depending on local resources and workflows, these components may be delivered as a multifactorial intervention in the ED or used to inform referral to further assessment and follow-up after discharge. Together, these findings support the ED not only as a point for case-finding, but also as a setting where fall-prevention interventions can be initiated and linked to ongoing care.

### Limitations

This review has several limitations. Not all outcomes could be meta-analysed due to limited study numbers or heterogeneous outcome measures. High heterogeneity in some analyses could not be resolved by sensitivity analyses, necessitating descriptive synthesis and vote counting. While vote counting does not account for effect size or study quality, it provides insight into consistency of effects when meta-analysis is not feasible. Many RCTs were judged at high risk of bias, largely due to low adherence, a common challenge in frail populations. Variability in adherence definitions, intervention intensity and follow-up likely underestimated intervention effects. Uptake of fall prevention is generally low, with ~7% of informed older adults engaging in intervention, suggesting that individual-level RCTs may underestimate real-world effectiveness [11, 62]. Alternative designs such as cluster or stepped-wedge randomised trials embedded in routine care minimising fall-specific framing as part of larger health research may better capture real-world effects and reduce behavioural bias [63–65].

## Conclusion

This systematic review and meta-analyses suggest that fall-prevention interventions initiated in the ED have favourable effects, particularly in reducing fall incidence among older adults. Although most secondary outcomes did not reach statistical significance, consistent positive trends were observed. Substantial heterogeneity, intervention variability and methodological challenges likely led to underestimation of true effects, underscoring the need for well-designed future studies.

## Supplementary Material

aa-26-0479-File002_afag227

## Implications for Practice and Research

Current evidence supports integrating multifactorial fall-prevention interventions into ED care pathways for older adults. Future research should identify patient subgroups most likely to benefit and determine which intervention components drive effectiveness. Pragmatic trial designs, including cluster and stepped-wedge RCTs, alongside implementation research, are needed to assess feasibility, cost-effectiveness and sustainability in real-world ED settings.
