# Supplementary material for: Emergency department-initiated interventions to reduce risk of recurrent falls in older adults: a systematic review and meta-analysis
Source: Age Ageing. 2026 Aug 3;55(8):afag227. doi: 10.1093/ageing/afag227 (PMC13430035; doi:10.1093/ageing/afag227)
Supplement: aa-26-0479-File002_afag227 [file aa-26-0479-file002_afag227.docx]

**Emergency department-initiated interventions to reduce risk of recurrent falls in older adults; a systematic review and meta-analyses**

**Supplement A. Search strategy**

**Ovid Medline Search Results – 23 Jun 2025**

| **Search** | **Query** | **Results** |
| --- | --- | --- |
| **#4** | **1 and 2 and 3** | **2,150** |
| **#3** | **exp "Aged"/ OR "Geroscience"/ OR "Geriatric Assessment"/ OR ("elderly" OR "eldest" OR "late life*" OR "frail" OR "frailty" OR "old age*" OR "oldest old*" OR "senior*" OR "senium" OR "very old*" OR "septuagenarian*" OR "octagenarian*" OR "octogenarian*" OR "nonagenarian*" OR "centarian*" OR "centenarian*" OR "supercentenarian*" OR "older people" OR "older subject*" OR "older patient*" OR "older age*" OR "older adult*" OR "older man" OR "older men" OR "older male*" OR "older woman" OR "older women" OR "older female*" OR "older population*" OR "older person*" OR "older individual*" OR "aged 60" OR "aged 65" OR "aged 70" OR "aged 75" OR "aged 80" OR "aged 90" OR "aged 100" OR "over 60" OR "over 65" OR "over 70" OR "over 75" OR "over 80" OR "over 90" OR "over 100" OR "60 years and older" OR "65 years and older" OR "70 years and older" OR "75 years and older" OR "80 years and older" OR "90 years and older" OR "65 years and over" OR "75 years and over" OR "80 years and over" OR "60 year and older" OR "65 year and older" OR "70 year and older" OR "60 years or older" OR "75 years or older" OR "80 years or older" OR "90 years or older" OR "65 years or over" OR "75 year or older" OR "older than 60" OR "older than 65" OR "older than 70" OR "older than 75" OR "older than 80" OR "older than 90" OR "geriat*" OR "gerontol*" or "geroscience*" or "gero-science*").ab,ti,kf.** | **4,099,639** |
| **#2** | **"Emergency Medical Services"/ or "Emergency Room Visits"/ or exp "Emergency Service, Hospital"/ or (("emergency" or "trauma" or "accident" or "acute care") adj3 ("center*" or "centre*" or "service*" or "room*" or "department*" or "ward*" or "unit*" or "setting*")).ab,ti,kf.** | **302,883** |
| **#1** | **exp "Accidental Falls"/ or (("fall*" or "trip*" or "slip*" or "stumbl*") adj10 ("prevent*" or "incidenc*" or "prevalence*" or "intervention*" or "hazard" or "risk*" or "assess*")).ab,ti,kf. or ("Accident Prevention"/ and ("fall*" or "trip*" or "slip*" or "stumbl*").ab,ti,kf.)** | **74,893** |

**Embase.com Search Results – 23 Jun 2025**

| **Search** | **Query** | **Results** |
| --- | --- | --- |
| **#5** | **#4 NOT ('conference abstract'/it OR 'conference review'/it) NOT 'clinical trial':dtype** | **3,595** |
| **#4** | **#1 AND #2 AND #3** | **4,411** |
| **#3** | **'aged'/exp OR 'gerontology'/exp OR 'geriatric assessment'/exp OR ("elderly" OR "eldest" OR "late life*" OR "frail" OR "frailty" OR "old age*" OR "oldest old*" OR "senior*" OR "senium" OR "very old*" OR "septuagenarian*" OR "octagenarian*" OR "octogenarian*" OR "nonagenarian*" OR "centarian*" OR "centenarian*" OR "supercentenarian*" OR "older people" OR "older subject*" OR "older patient*" OR "older age*" OR "older adult*" OR "older man" OR "older men" OR "older male*" OR "older woman" OR "older women" OR "older female*" OR "older population*" OR "older person*" OR "older individual*" OR "aged 60" OR "aged 65" OR "aged 70" OR "aged 75" OR "aged 80" OR "aged 90" OR "aged 100" OR "over 60" OR "over 65" OR "over 70" OR "over 75" OR "over 80" OR "over 90" OR "over 100" OR "60 years and older" OR "65 years and older" OR "70 years and older" OR "75 years and older" OR "80 years and older" OR "90 years and older" OR "65 years and over" OR "75 years and over" OR "80 years and over" OR "60 year and older" OR "65 year and older" OR "70 year and older" OR "60 years OR older" OR "75 years OR older" OR "80 years OR older" OR "90 years OR older" OR "65 years OR over" OR "75 year OR older" OR "older than 60" OR "older than 65" OR "older than 70" OR "older than 75" OR "older than 80" OR "older than 90" OR "geriat*" OR "gerontol*" OR "geroscience*" OR "gero-science*"):ab,ti,kw** | **4,930,345** |
| **#2** | **'emergency health service'/de OR 'emergency department visit'/exp OR 'hospital emergency service'/exp OR 'emergency ward'/exp OR (("emergency" or "trauma" or "accident" or "acute care") NEAR/3 ("center*" or "centre*" or "service*" or "room*" or "department*" or "ward*" or "unit*" or "setting*")):ab,ti,kw** | **500,637** |
| **#1** | **'falling'/exp OR 'fall prevention'/exp OR (("fall*" OR "trip*" OR "slip*" OR "stumbl*") NEAR/10 ("prevent*" OR "incidenc*" OR "prevalence*" OR "intervention*" OR "hazard" OR "risk*" OR "assess*")):ab,ti,kw OR ('accident prevention'/exp AND ("fall*" OR "trip*" OR "slip*" OR "stumbl*"):ab,ti,kw)** | **128,024** |

**CINAHL (Ebsco) Search Results – 23 Jun 2025**

| **Search** | **Query** | **Results** |
| --- | --- | --- |
| **S4** | **S1 AND S2 AND S3** | **1,470** |
| **S3** | **(MH "Aged") OR (MH "Aged, 80 and Over") OR (MH "Centenarians") OR (MH "Nonagenarians") OR (MH "Octogenarians") OR (MH "Hospitalization of Older Persons") OR (MH "Frail Elderly") OR (MH "Geroscience") OR (MH "Geriatric Assessment+") OR TI ("elderly" OR "eldest" OR "late life*" OR "frail" OR "frailty" OR "old age*" OR "oldest old*" OR "senior*" OR "senium" OR "very old*" OR "septuagenarian*" OR "octagenarian*" OR "octogenarian*" OR "nonagenarian*" OR "centarian*" OR "centenarian*" OR "supercentenarian*" OR "older people" OR "older subject*" OR "older patient*" OR "older age*" OR "older adult*" OR "older man" OR "older men" OR "older male*" OR "older woman" OR "older women" OR "older female*" OR "older population*" OR "older person*" OR "older individual*" OR "aged 60" OR "aged 65" OR "aged 70" OR "aged 75" OR "aged 80" OR "aged 90" OR "aged 100" OR "over 60" OR "over 65" OR "over 70" OR "over 75" OR "over 80" OR "over 90" OR "over 100" OR "60 years and older" OR "65 years and older" OR "70 years and older" OR "75 years and older" OR "80 years and older" OR "90 years and older" OR "65 years and over" OR "75 years and over" OR "80 years and over" OR "60 year and older" OR "65 year and older" OR "70 year and older" OR "60 years OR older" OR "75 years OR older" OR "80 years OR older" OR "90 years OR older" OR "65 years OR over" OR "75 year OR older" OR "older than 60" OR "older than 65" OR "older than 70" OR "older than 75" OR "older than 80" OR "older than 90" OR "geriat*" OR "gerontol*" OR "geroscience*" OR "gero-science*") OR AB ("elderly" OR "eldest" OR "late life*" OR "frail" OR "frailty" OR "old age*" OR "oldest old*" OR "senior*" OR "senium" OR "very old*" OR "septuagenarian*" OR "octagenarian*" OR "octogenarian*" OR "nonagenarian*" OR "centarian*" OR "centenarian*" OR "supercentenarian*" OR "older people" OR "older subject*" OR "older patient*" OR "older age*" OR "older adult*" OR "older man" OR "older men" OR "older male*" OR "older woman" OR "older women" OR "older female*" OR "older population*" OR "older person*" OR "older individual*" OR "aged 60" OR "aged 65" OR "aged 70" OR "aged 75" OR "aged 80" OR "aged 90" OR "aged 100" OR "over 60" OR "over 65" OR "over 70" OR "over 75" OR "over 80" OR "over 90" OR "over 100" OR "60 years and older" OR "65 years and older" OR "70 years and older" OR "75 years and older" OR "80 years and older" OR "90 years and older" OR "65 years and over" OR "75 years and over" OR "80 years and over" OR "60 year and older" OR "65 year and older" OR "70 year and older" OR "60 years OR older" OR "75 years OR older" OR "80 years OR older" OR "90 years OR older" OR "65 years OR over" OR "75 year OR older" OR "older than 60" OR "older than 65" OR "older than 70" OR "older than 75" OR "older than 80" OR "older than 90" OR "geriat*" OR "gerontol*" OR "geroscience*" OR "gero-science*")** | **1,123,244** |
| **S2** | **MH "Emergency Medical Services+") OR (MH "Emergency Service+") OR (MH "Emergency Room Visits") OR TI (("emergency" or "trauma" or "accident" or "acute care") N3 ("center*" or "centre*" or "service*" or "room*" or "department*" or "ward*" or "unit*" or "setting*")) OR AB (("emergency" or "trauma" or "accident" or "acute care") N3 ("center*" or "centre*" or "service*" or "room*" or "department*" or "ward*" or "unit*" or "setting*"))** | **185,184** |
| **S1** | **(MH "Accidental Falls") OR TI (("fall*" OR "trip*" OR "slip*" OR "stumbl*") N10 ("prevent*" OR "incidenc*" OR "prevalence*" OR "intervention*" OR "hazard" OR "risk*" OR "assess*")) OR AB (("fall*" OR "trip*" OR "slip*" OR "stumbl*") N10 ("prevent*" OR "incidenc*" OR "prevalence*" OR "intervention*" OR "hazard" OR "risk*" OR "assess*")) OR**  **((MH "Safety") AND (TI ("fall*" OR "trip*" OR "slip*" OR "stumbl*") OR AB ("fall*" OR "trip*" OR "slip*" OR "stumbl*")))** | **41,933** |

**PEDro (Physiotherapy Evidence Database) Search Results – 23 Jun 2025**

| **Search** | **Query** | **Results** |
| --- | --- | --- |
| **#1** | **Abstract & Title: fall* AND prevent* AND emergen*** | **34** |

**Web of Science (Core Collection) Search Results – 23 Jun 2025**

| **Search** | **Query** | **Results** |
| --- | --- | --- |
| **#4** | **#1 AND #2 AND #3** | **1,190** |
| **#3** | **TS=("elderly" OR "eldest" OR "late life*" OR "frail" OR "frailty" OR "old age*" OR "oldest old*" OR "senior*" OR "senium" OR "very old*" OR "septuagenarian*" OR "octagenarian*" OR "octogenarian*" OR "nonagenarian*" OR "centarian*" OR "centenarian*" OR "supercentenarian*" OR "older people" OR "older subject*" OR "older patient*" OR "older age*" OR "older adult*" OR "older man" OR "older men" OR "older male*" OR "older woman" OR "older women" OR "older female*" OR "older population*" OR "older person*" OR "older individual*" OR "aged 60" OR "aged 65" OR "aged 70" OR "aged 75" OR "aged 80" OR "aged 90" OR "aged 100" OR "over 60" OR "over 65" OR "over 70" OR "over 75" OR "over 80" OR "over 90" OR "over 100" OR "60 years and older" OR "65 years and older" OR "70 years and older" OR "75 years and older" OR "80 years and older" OR "90 years and older" OR "65 years and over" OR "75 years and over" OR "80 years and over" OR "60 year and older" OR "65 year and older" OR "70 year and older" OR "60 years OR older" OR "75 years OR older" OR "80 years OR older" OR "90 years OR older" OR "65 years OR over" OR "75 year OR older" OR "older than 60" OR "older than 65" OR "older than 70" OR "older than 75" OR "older than 80" OR "older than 90" OR "geriat*" OR "gerontol*" OR "geroscience*" OR "gero-science*")** | **1,170,847** |
| **#2** | **TS=(("emergency" or "trauma" or "accident" or "acute care") NEAR/3 ("center*" or "centre*" or "service*" or "room*" or "department*" or "ward*" or "unit*" or "setting*"))** | **264,704** |
| **#1** | **TS=(("fall*" OR "trip*" OR "slip*" OR "stumbl*") NEAR/10 ("prevent*" OR "incidenc*" OR "prevalence*" OR "intervention*" OR "hazard" OR "risk*" OR "assess*"))** | **79,342** |

**Scopus Search Results – 23 Jun 2025**

| **Search** | **Query** | **Results** |
| --- | --- | --- |
| **#4** | **#1 AND #2 AND #3** | **1,185** |
| **#3** | **TITLE-ABS ("elderly" OR "eldest" OR "late life*" OR "frail" OR "frailty" OR "old age*" OR "oldest old*" OR "senior*" OR "senium" OR "very old*" OR "septuagenarian*" OR "octagenarian*" OR "octogenarian*" OR "nonagenarian*" OR "centarian*" OR "centenarian*" OR "supercentenarian*" OR "older people" OR "older subject*" OR "older patient*" OR "older age*" OR "older adult*" OR "older man" OR "older men" OR "older male*" OR "older woman" OR "older women" OR "older female*" OR "older population*" OR "older person*" OR "older individual*" OR "aged 60" OR "aged 65" OR "aged 70" OR "aged 75" OR "aged 80" OR "aged 90" OR "aged 100" OR "over 60" OR "over 65" OR "over 70" OR "over 75" OR "over 80" OR "over 90" OR "over 100" OR "60 years and older" OR "65 years and older" OR "70 years and older" OR "75 years and older" OR "80 years and older" OR "90 years and older" OR "65 years and over" OR "75 years and over" OR "80 years and over" OR "60 year and older" OR "65 year and older" OR "70 year and older" OR "60 years OR older" OR "75 years OR older" OR "80 years OR older" OR "90 years OR older" OR "65 years OR over" OR "75 year OR older" OR "older than 60" OR "older than 65" OR "older than 70" OR "older than 75" OR "older than 80" OR "older than 90" OR "geriat*" OR "gerontol*" OR "geroscience*" OR "gero-science*") OR AUTHKEY ("elderly" OR "eldest" OR "late life*" OR "frail" OR "frailty" OR "old age*" OR "oldest old*" OR "senior*" OR "senium" OR "very old*" OR "septuagenarian*" OR "octagenarian*" OR "octogenarian*" OR "nonagenarian*" OR "centarian*" OR "centenarian*" OR "supercentenarian*" OR "older people" OR "older subject*" OR "older patient*" OR "older age*" OR "older adult*" OR "older man" OR "older men" OR "older male*" OR "older woman" OR "older women" OR "older female*" OR "older population*" OR "older person*" OR "older individual*" OR "aged 60" OR "aged 65" OR "aged 70" OR "aged 75" OR "aged 80" OR "aged 90" OR "aged 100" OR "over 60" OR "over 65" OR "over 70" OR "over 75" OR "over 80" OR "over 90" OR "over 100" OR "60 years and older" OR "65 years and older" OR "70 years and older" OR "75 years and older" OR "80 years and older" OR "90 years and older" OR "65 years and over" OR "75 years and over" OR "80 years and over" OR "60 year and older" OR "65 year and older" OR "70 year and older" OR "60 years OR older" OR "75 years OR older" OR "80 years OR older" OR "90 years OR older" OR "65 years OR over" OR "75 year OR older" OR "older than 60" OR "older than 65" OR "older than 70" OR "older than 75" OR "older than 80" OR "older than 90" OR "geriat*" OR "gerontol*" OR "geroscience*" OR "gero-science*")** | **1,347,421** |
| **#2** | **TITLE-ABS (("emergency" or "trauma" or "accident" or "acute care") W/3 ("center*" or "centre*" or "service*" or "room*" or "department*" or "ward*" or "unit*" or "setting*")) OR AUTHKEY (("emergency" or "trauma" or "accident" or "acute care") W/3 ("center*" or "centre*" or "service*" or "room*" or "department*" or "ward*" or "unit*" or "setting*"))** | **296,941** |
| **#1** | **TITLE-ABS (("fall*" OR "trip*" OR "slip*" OR "stumbl*") W/10 ("prevent*" OR "incidenc*" OR "prevalence*" OR "intervention*" OR "hazard" OR "risk*" OR "assess*")) OR AUTHKEY (("fall*" OR "trip*" OR "slip*" OR "stumbl*") W/10 ("prevent*" OR "incidenc*" OR "prevalence*" OR "intervention*" OR "hazard" OR "risk*" OR "assess*"))** | **104,324** |

**Supplement B. Study Quality Assessment**

| **NIH_CT_ Item →** | **1** | **2** | **3** | **4** | **5** | **6** | **7** | **8** | **9** | **10** | **11** | **12** | **13** | **14** | **Total score** |
| --- | --- | --- | --- | --- | --- | --- | --- | --- | --- | --- | --- | --- | --- | --- | --- |
| **RCT ↓** |  |  |  |  |  |  |  |  |  |  |  |  |  |  |  |
| **Barker 2018 (26)** | Y | Y | Y | N | Y | Y | N | N | Y | Y | Y | N | Y | Y | **10** |
| **Benhamou 2025 (27)** | Y | Y | N | N | N | N | N | N | N | Y | Y | N | Y | N | **5** |
| **Boyé 2017 (29), Polinder 2016 (46)** | Y | Y | Y | N | CD/NA | Y | Y | Y | Y | Y | Y | N | Y | Y | **11** |
| **Chu 2016 (30)** | Y | Y | Y | N | Y | Y | Y | Y | N | CD/NA | Y | Y | Y | Y | **11** |
| **Close 1999 (32)** | Y | Y | Y | N | N | Y | N | Y | N | Y | Y | Y | Y | CD/NA | **9** |
| **Dadgari 2022 (33)** | Y | Y | Y | N | CD/NA | Y | Y | Y | Y | Y | N | N | Y | Y | **10** |
| **Davison 2005 (34)** | Y | Y | Y | N | Y | Y | Y | Y | Y | Y | Y | Y | Y | Y | **13** |
| **Goldberg 2020 (37)** | Y | Y | N | N | Y | Y | Y | CD/NA | Y | Y | Y | N | Y | Y | **10** |
| **Harper 2017 (38)** | Y | Y | Y | N | N | Y | N | N | Y | Y | Y | N | N | Y | **8** |
| **Hendriks 2008 (40)** | Y | Y | Y | N | Y | Y | N | Y | N | Y | Y | Y | Y | Y | **11** |
| **Lightbody 2002 (43)** | Y | Y | N | N | N | Y | N | N | N | CD/NA | Y | Y | N | Y | **6** |
| **Matchar 2017 (44), Matchar 2019 (45)** | Y | Y | Y | N | Y | Y | Y | Y | Y | Y | Y | Y | Y | Y | **13** |
| **Russell 2010 (48)** | Y | Y | Y | N | Y | Y | Y | Y | N | Y | Y | Y | Y | Y | **12** |
| **Shaw 2003 (49)** | Y | Y | Y | N | Y | Y | Y | Y | Y | Y | Y | Y | Y | Y | **13** |
| **Vind 2009 (51), Vind 2010 (50)** | Y | Y | Y | N | Y | Y | Y | Y | Y | Y | Y | Y | N | Y | **12** |
| **de Vries**  **2010 (35)** | Y | Y | Y | N | CD/NA | Y | N | Y | N | Y | Y | Y | Y | Y | **10** |
| **Whitehead 2003 (52)** | Y | Y | Y | N | Y | N | Y | Y | Y | Y | Y | N | Y | Y | **11** |
| **NIH_CT_ Items:**   1. Was the study described as randomized, a randomized trial, a randomized clinical trial, or an RCT? 2. Was the method of randomization adequate (i.e., use of randomly generated assignment)? 3. Was the treatment allocation concealed (so that assignments could not be predicted)? 4. Were study participants and providers blinded to treatment group assignment? 5. Were the people assessing the outcomes blinded to the participants' group assignments? 6. Were the groups similar at baseline on important characteristics that could affect outcomes (e.g., demographics, risk factors, co-morbid conditions)? 7. Was the overall drop-out rate from the study at endpoint 20% or lower of the number allocated to treatment? 8. Was the differential drop-out rate (between treatment groups) at endpoint 15 percentage points or lower? 9. Was there high adherence to the intervention protocols for each treatment group? 10. Were other interventions avoided or similar in the groups (e.g., similar background treatments)? 11. Were outcomes assessed using valid and reliable measures, implemented consistently across all study participants? 12. Did the authors report that the sample size was sufficiently large to be able to detect a difference in the main outcome between groups with at least 80% power? 13. Were outcomes reported or subgroups analysed prespecified (i.e., identified before analyses were conducted)? 14. Were all randomized participants analysed in the group to which they were originally assigned, i.e., did they use an intention-to-treat analysis? | | | | | | | | | | | | | | |  |
| N: no; Y: Yes, CD: cannot determine; NA: not applicable; NR: not reported | | | | | | | | | | | | | | |  |

| **NIH_CT_ Item →** | **1** | **2** | **3** | **4** | **5** | **6** | **7** | **8** | **9** | **10** | **11** | **12** | **13** | **14** | **Total score** |
| --- | --- | --- | --- | --- | --- | --- | --- | --- | --- | --- | --- | --- | --- | --- | --- |
| **Non-RCT↓** |  |  |  |  |  |  |  |  |  |  |  |  |  |  |  |
| **Bogucki**  **2023 (28)** | N | CD/NA | N | N | CD/NA | CD/NA | N | Y | N | CD/NA | Y | CD/NA | Y | N | **3** |
| **Clementz**  **2019 (31)** | N | CD/NA | N | N | CD/NA | CD/NA | Y | Y | Y | CD/NA | Y | N | Y | Y | **6** |
| **Fulbrook 2025 (36)** | N | Y | N | N | CD/NA | Y | Y | Y | Y | Y | Y | N | Y | Y | **9** |
| **Harper**  **2013 (39)** | N | CD/NA | CD/NA | N | N | N | Y | Y | N | Y | Y | Y | Y | Y | **7** |
| **Hepkema**  **2022 (15)** | N | CD/NA | CD/NA | N | N | N | N | N | Y | CD/NA | Y | N | CD/NA | N | **2** |
| **Leahy**  **2014 (42)** | N | CD/NA | N | N | CD/NA | N | N | CD/NA | N | CD/NA | CD/NA | N | Y | N | **1** |

| **NIH_PP_ Item →** | **1** | **2** | **3** | **4** | **5** | **6** | **7** | **8** | **9** | **10** | **11** | **12** | **Total score** |
| --- | --- | --- | --- | --- | --- | --- | --- | --- | --- | --- | --- | --- | --- |
| **Pre-post↓** |  |  |  |  |  |  |  |  |  |  |  |  |  |
| **Ageron**  **2016 (24)** | Y | Y | Y | Y | Y | N | Y | CD/NA | Y | Y | N | Y | **9** |
| **Baraff**  **1999 (25)** | Y | Y | Y | Y | Y | CD/NA | N | N | N | Y | N | CD/NA | **6** |
| **Jusmanova 2021 (41)** | Y | Y | Y | Y | Y | CD/NA | Y | N | CD/NA | Y | N | CD/NA | **7** |
| **Rosario**  **2023 (47)** | N | N | Y | CD/NA | CD/NA | N | N | N | CD/NA | Y | CD/NA | CD/NA | **2** |
| **NIH_PP_ Items:**   1. Was the study question or objective clearly stated? 2. Were eligibility/selection criteria for the study population prespecified and clearly described? 3. Were the participants in the study representative of those who would be eligible for the test/service/intervention in the general or clinical population of interest? 4. Were all eligible participants that met the prespecified entry criteria enrolled? 5. Was the sample size sufficiently large to provide confidence in the findings? 6. Was the test/service/intervention clearly described and delivered consistently across the study population? 7. Were the outcome measures prespecified, clearly defined, valid, reliable, and assessed consistently across all study participants? 8. Were the people assessing the outcomes blinded to the participants' exposures/interventions? 9. Was the loss to follow-up after baseline 20% or less? Were those lost to follow-up accounted for in the analysis? 10. Did the statistical methods examine changes in outcome measures from before to after the intervention? Were statistical tests done that provided p values for the pre-to-post changes? 11. Were outcome measures of interest taken multiple times before the intervention and multiple times after the intervention (i.e., did they use an interrupted time-series design)? 12. If the intervention was conducted at a group level (e.g., a whole hospital, a community, etc.) did the statistical analysis take into account the use of individual-level data to determine effects at the group level? | | | | | | | | | | | | |  |
| N: no; Y: Yes, CD: cannot determine; NA: not applicable; NR: not reported | | | | | | | | | | | | |  |

**Supplement C. Study Risk of Bias Assessments**

| *RoB2 Theme →* | *Randomisation* | *Assignment to interventions* | *Adherence to interventions* | *Missing data* | *Measurement of outcome* | *Reported result* | ***Overall*** |
| --- | --- | --- | --- | --- | --- | --- | --- |
| **RCT ↓** |  |  |  |  |  |  |  |
| **Barker 2018 (26)** | Low risk | Low risk | Low risk | Low risk | Low risk | Low risk | Low risk |
| **Benhamou 2025 (27)** | High risk | High risk | High risk | Low risk | Low risk | Some concerns | High risk |
| **Boyé 2017 (29), Polinder 2016 (46)** | Low risk | Low risk | Low risk | Low risk | Low risk | Low risk | Low risk |
| **Chu 2016 (30)** | Low risk | Low risk | High risk | High risk | Low risk | Some concerns | High risk |
| **Close 1999 (32)** | Low risk | High risk | High risk | Low risk | Low risk | Some concerns | High risk |
| **Dadgari 2022 (33)** | Low risk | Some concerns | High risk | Some concerns | High risk | Some concerns | High risk |
| **Davison 2005 (34)** | Some concerns | Low risk | High risk | Low risk | Low risk | Some concerns | High risk |
| **Goldberg 2020 (37)** | High risk | Low risk | Low risk | Low risk | Low risk | Low risk | High risk |
| **Harper 2017 (38)** | Low risk | High risk | High risk | Low risk | Low risk | Some concerns | High risk |
| **Hendriks 2008 (40)** | Low risk | Low risk | High risk | Low risk | Low risk | Low risk | High risk |
| **Lightbody 2002 (43)** | Some concerns | High risk | High risk | Low risk | Low risk | Some concerns | High risk |
| **Matchar 2017 (44), Matchar 2019 (45)** | Low risk | Low risk | Low risk | Low risk | Low risk | Some concerns | Some concerns |
| **Russell 2010 (48)** | Low risk | High risk | High risk | Low risk | Low risk | Some concerns | High risk |
| **Shaw 2003 (49)** | Low risk | Low risk | Some concerns | Low risk | Low risk | Some concerns | Some concerns |
| **Vind 2009 (51), Vind 2010 (50)** | Low risk | Low risk | Low risk | Low risk | Low risk | Low risk | Low risk |
| **de Vries 2010 (35)** | Low risk | Low risk | Some concerns | Low risk | Some concerns | Low risk | Some concerns |
| **Whitehead 2003 (52)** | Some concerns | Low risk | Low risk | Low risk | Low risk | Some concerns | Some concerns |

| *ROBINS-I Theme →* | *Preliminary considerations* | *Confounding* | *Classification of intervention* | *Selection of participants* | *Deviations from intended interventions* | *Missing data* | *Measurement of outcome* | *Selection of reported result* | ***Overall*** |
| --- | --- | --- | --- | --- | --- | --- | --- | --- | --- |
| **Non-RCT ↓** |  |  |  |  |  |  |  |  |  |
| **Bogucki**  **2023 (28)** | No issue | Moderate | Low | Serious | Low | Serious | Serious | Low | Serious |
| **Clementz**  **2019 (31)** | No issue | Low | Low | Low | Low | Serious | Low | Low | Serious |
| **Fulbrook 2025 (36)** | No issue | Moderate | Low | Low | Low | Serious | Low | Low | Serious |
| **Harper**  **2013 (39)** | Critical | NA | NA | NA | NA | NA | NA | NA | Critical |
| **Hepkema**  **2022 (15)** | No issue | NA | Low | Low | NA | Low | Low | Low | Low* |
| **Leahy**  **2014 (42)** | No issue | Moderate | Low | Low | Moderate | Serious | Low | Low | Serious |

| ROBINS-I Theme → | *Preliminary considerations* | *Confounding* | *Classification of intervention* | *Selection of participants* | *Deviations from intended interventions* | *Missing data* | *Measurement of outcome* | *Selection of reported result* | ***Overall*** |
| --- | --- | --- | --- | --- | --- | --- | --- | --- | --- |
| **Pre-post study ↓** |  |  |  |  |  |  |  |  |  |
| **Ageron**  **2016 (24)** | No issue | Low | Low | Low | Low | Low | Low | Low | Low |
| **Baraff**  **1999 (25)** | Critical* | Moderate | Low | Low | Moderate | Critical | Low | Low | Critical |
| **Jusmanova 2021 (41)** | No issue | Serious | Low | Low | Serious | Low | Low | Low | Serious |
| **Rosario**  **2023 (47)** | Critical | NA | NA | NA | NA | NA | NA | NA | Critical |

**Supplement D. Outcome measures during follow-up.**

| Author | Number of falls & time to first fall | Nr of patients with one or more falls (fallers) | Fractures and injury | ED revisits | Hospital admissions | Mortality, QoL & cost | Physical functioning, participation, Concerns about falling |
| --- | --- | --- | --- | --- | --- | --- | --- |
| Randomised Controlled Trials | | | | | | | |
| Barker 2018 (1) | Nr of falls  I: 220, C: 355,  Fall rate per person years  I: 1.15, C: 1.83, IRR: 0.65 (95% CI 0.43–0.99); p 0.042 | Fallers  I: 100 (46%) C: 106 (50%)  Recurrent fallers (≥ 3)  I: 47 (22%); C 58 (27%) | Injurious falls : I: 112 (51%) C: 172 (49%)  Rate per person years IRR 0.81 (0.51–1.29) p 0.374  Fractures: I: 10 (5%), C: 23 (9%)  Rate per person years  I: 0.05, C: 0.12, IRR 0.37 (95% CI 0.15–0.91); p 0.03  Hip fractures: none | AC ED revisits  I: 141, C: 154  Rate per person years  I:0.05, C: 0.12. IRR 0.92 (95%CI 0.64–1.32), p 0.653 | AC hospital admissions  C: I: 173, C: 226  Rate per person years  I: 0.9, C: 1.2; IRR 0.78 (0.55 –1.10), p 0.152 | Death during FU  I: 2 (1%), C: 1 (0.5%)  EQ5D  Overall health state Δ 2.25 (95%CI -1.13-5.64) p 0.190  Utility score Δ  0.03 (95%CI -0.02-0.08) p 0.231 | EQ5D mobility Δ  -0.04 (-0.14 - 0.06) p 0.421  EQ5D usual activity Δ -0.09 (95%CI -0.19 - -0.008) p 0.076  Concerns about falling  Short FES-I Δ -0.70 (95%CI -1.84-0.43) p 0.224 |
| Benhamou 2025 (2) |  | Fallers  I: 3 (6.8%) C: 7 (11.2%), p 0.482 |  |  |  | SPPB, median (IQR)  day 7  I: 8.0 (7.0–9.0), C: 8.0 (4.3–10), p 0.636 | Short FES-I, median (IQR)  Baseline I: 10 (8.0–12), C: 9.0 (7.0–10)  7 days: I: 10 (8.0-13), C: 10 (8.0-13)  42 days: I: 8 (7–9.3), C: 7 (7–9) |
| Boyé 2017, Polinder 2016 (3, 4) | Time to first fall:  HR 1.17 (95% CI 0.89 - 1.54)  Time to second fall:  HR 1.19 (95%CI 0.78–1.82) | Fallers  I: 115 (37%), C 91 (34%), p 0.33  Recurrent fallers (≥2)  I: 50 (16%), C: 38 (14%), p 0.45 | - | Time to first FR ED revisit  HR 0.85 (95%CI 0.43–1.68)  Time to first fall-related GP-visit HR 0.66 (95%CI 0.42–1.06); | - | Death during FU: I: 1 C: 2  EQ-5D change in 12 months (SD): I: 0.01 (0.24) C: -0.04 (0.22) p 0.02  FR health care costs I: 2324 EUR, C: 2285 p > 0.05 | SF-12 PCS score change (SD)  I: -2.6 (8.5), C: -3.9 (8.5) p 0.08 |
| Chu 2016 (5) | Nr of falls  6 months:  I: 3, C: 12, p 0.02  12 months:  I: 16, C: 30, p 0.14  Time to first fall (days, SD) I: 242 (113), C: 174 (122), p 0.11 | Fallers  6 months: I: 3, C: 12 (p 0.03).  12 months: I: 13, C: 21, p 0.26  Recurrent fallers (≥2)  12 months: I: 2, C 6, p 0.28 | - | Fall related ED revisits  I: 13, C: 20, p 0.40 | Fall related hospital admissions:  I: 4 (4%), C: 6 (6%) | Death during FU  I: 3 C: 3 | Modified barthel index (SD)  I 47 (5), C: 47 (5) p 0.64.  FAI; I 19(8), C 20 (8) |
| Close 1999 (6) | Nr of falls  I: 183 falls, C: 510 falls  Median number of falls (IQR); I: 0 (0–3), C: 1 (0–3) | Fallers  I: 59 (32%), C 111 (52%), OR 0.39 (95% CI 0.23–0.66)  Recurrent fallers (≥3)  I: 21 (11%), C: 55 (26%), OR 0.3 (95% CI 0.2–0.7) | Injurious fallers  I: 8 (4%), C: 16 (8%), p 0.26 | - | AC hospital admissions:  I: 69, C: 97; OR 0.61 (95%CI 0.35–1.05) | Death during FU  I 19 (10%), C 27 (13%) | Barthel index score (SD)  I: 19 (3), C: 17 (4), change in scores over time: p<0·0001  Able to go out alone  I: 108 (77%), C: 106 (65%) |
| Dadgari 2022 (7) | Recurrent falls (SD)  I: 0.16 (0.17), C: 0.40 (0.37), mean Δ: −0.24 (95% CI −0.43−0.049), p 0.014 | Fallers  I: 8 (16%), C: 16 (32%) p 0.014 | Injury Severity Score (SD)  I: 2.0 (0.8), C: 3.2 (2.3), mean difference−1.2 (−1.9-−0.5), p 0.001 | - | - | - | ADL I: 1.4 (SD 0.5), C: 1.5 (SD 0.5), p 0.422 |
| Davison 2005 (8) | Nr of falls  I: 387, C: 617, RR: 0.64 (95%CI 0.46 - 0.90) | Fallers  I: 94 (65%), C: 102 (68%), RR: 0.95 (95%CI 0.81-1.12) | Hip fractures  I: 1, C: 2; RR: 0.48 (95% CI 0.04 - 5.29)  Other fractures  I: 6 (4%), C: 11 (7%), RR: 0.53 (0.2-1.39) | Fall related ED revisits:  I: 25 (16%), C: 27 (18%) | Fall related hospital admission: I: 14 (9%)  C: 17 (11%) | Death during FU  I: 3 (2%), C: 5 (3%), RR: 0.58 (95% CI 0.14 - 2.38) | Mean Balance Score (SD)  I: 61 (28), C: 53 (29)  Mean Δ: 7.5 (95%CI 0.72-14.2) |
| Goldberg 2020 (9) | - | - | - | FR ED revisits:I: 9/319 C: 24/297 person months  Participants with one or more FR ED revisits; I: 8 (15%), C: 15 (27%). aIRR 0.34 (95% CI 0.15-0.76)  AC ED revisits: I: 30/319; C: 66/297 person months  Participants with one or more ED revisits: I: 20 (36%), C: 29 (53%), aIRR 0.47 (0.29–0.74) | FR hospital admissions:  I: 7/319 person months; C: 6/297 person months  Part. with ≥1 FR hospital admissions: I: 6 (11%), C: 6 (11%). aIRR: 0.99 (0.31–3.27)  AC hospital admissions: I: 19/319 person months, C: 34/297 person months  Part. with ≥1 AC hospital admissions  I: 13 (23%), C: 21 (38%) aIRR: 0.57 (0.31–1.04) | Death during FU  I: 3/55 (5%), C: 10/55 (18%) | - |
| Harper 2017 (10) | Time to first fall  No difference between groups, p 0.410 | Fallers  I: 11 (21%), C: 8 (14%), p 0.373 | - | - | - | Death during FU  I: 1, C: 1 | FIM/FAM score:  I: 74.6  C: 74.7 |
| Hendriks 2008 (11) | Time to first fall  Total Follow up (1–12 mo), HR 1.08, p 0.66;  After programme implementation (5–12 mo), HR 0.79, p 0.30 | Fallers  4 months: I: 42 (31%), C: 37 (26%); OR 1.36 (95% CI 0.77–2.41), p 0.29  12 months: I: 55 (46%) C: 61 (41%), OR 0.86 (95%CI 0.50-1.49) p 0.59  Recurrent fallers  4 months: I: 14 (10%), C: 16 (11%); OR 0.91 (95%CI 0.39–2.11), p 0.83  12 months: I: 32 (26%), C: 34 (26%), OR 0.95 (95% CI 0.51-1.78) | Injurious falles  4 months  I: 10 (8%), C: 14 (11%) p 0.62; OR 0.79 (95%CI 0.31–2.00)  12 months  I: 14 (15%) C: 20 (21%) p 0.53, OR 0.77 (95% CI 0.35-1.73) | - | - | Death during FU  I: 5, C: 1  EuroQol  I: 0.70, C: 0.71, B -0.012 (-0.06 to 0.03), p 0.59 | FAI (SD): I: 26 (8) C: 25 (9); B 0.37 (95%CI -0.90-1.63), p 0.57  ADL (SD): I: 15 (2) 15 (6); B -0.03 (-0.64-0.64), p 0.94.  Activity avoidance: I: 55 (45%), C: 48 (36%), OR 1.57 (95%CI 0.84–2.97) p 0.16  Social participation (SD)  I: 6 (2), C: 6 (2.0), B - 0.07 (955%CI 0.52-0.37) p 0.75  Concerns about falling: I: 79 (64%), C: 81 (60), OR 1.31 (0.69-2.5), p 0.42 |
| Lightbody 2002 (12) | Nr of falls  I: 141, C: 171 | Fallers  I: 39 (25%), C: 41 (26%), p 1.00 | - | Fall related ED revisits:  I: 43, C: 58, p 0.82 | Fall related hospital admissions: I: 8, C: 10, p 0.87 | Death during FU  I: 11 C: 7 | Barthel index (mean, SD): I: 18.5 (2.4), C: 17.8 (3.6), p < 0.04  Life Space Diameter (median, IQR):  I: 7 (5-7), C: 6 (4-7), p < 0.02 |
| Matchar 2017, Matchar 2019 (13, 14) | - | Fallers  I: 54 (31%) C: 67 (38%), OR 0.72 (95% CI 0.46–1.12) | Injurious fallers  I: 25 (14%) C: 40 (23%)  OR 0.56 (95% CI, 0.32–0.98) | - | - | Death during FU  I: 6 C: 7  QoL: Gain of 0.003 QALYs in intervention group compared to control group | Physical performance (SPPB score)  Less deterioration in I group compared to C group; mean Δ: 0.6, p 0.029 |
| Russell 2010 (15) | Nr of falls  I: 908, C: 1449  Falls/person-year  I: 2.77, C: 4.24  aRR 0.87 (95%CI 0.65–1.17) | Fallers  I: 163 (51%), C: 151 (46%), RR: 1.11 (95%CI 0.95-1.31) | Injurious fallers  I: 118 (37%), C: 115 (35 %), RR 1.06 (95%CI 0.86 - 1.29)  Peripheral fracture  I: 8 (2.5), C: 15 (4.6); RR: 0.55 (95% 0.24–1.28) | Fall related ED revisits  per person year  I: 57 (0.18) C: 58 (0.18) RR 1.03 (0.68-1.54)  AC ED revisits  per person year  I: 221 (0.71) C: 210 (0.64). RR 1.13 (0.83-1.52) | Fall related days in hospital: I: 622 (rate 1.99); C: 396 (rate 1.20); iRR 1.29 (0.75–2.22)  AC days in hospital; I: 1526 (rate 4.9); C: 1524 (rate 4.7); RR 1.29 (0.75–2.22) | Death during FU  7 in both groups | - |
| Shaw 2003 (16) | Nr of falls  I: 652, C: 728, RR: -0.2 (95%CI -0.32 - 0.09)  Time to first fall weeks, median (IQR)  I: 11 (2-41), C: 11 (2-33), p 0.459 | Fallers  I: 96 (74%), C: 115 (80%), RR 0.92 (95% CI 0.81 - 1.05) | Major injury rate  I: 37 (28%), C 31 (21%), RR: 1.32 (95%CI 0.87 - 2.00)  Hip fractures  I: 6 (5%), C: 12 (8%), RR: 0.55 (0.21-1.43) | Fall related ED revisits:  I: 52 (40%), C: 46 (32%)  RR 1.25 (95%CI 0.91-1.72) | Fall related hospital admission:  I: 19 (15%) C: 19 (13%)  RR: 1.11 (95%CI 0.61- 2.00) | Death during FU  I: 27 (21%), C: 29 (20%), RR: 1.03 (95% CI: 0.65-1.64) | - |
| Vind 2009, Vind 2010 (17, 18) | Nr of falls:  I: 422, C: 398, RR 1.06 (95%CI 0.75–1.51)  Time to first fall: HR 1.11 (95% CI 0.84–1.45) | Fallers  I: 110 (56%), C: 101 (51%), OR: 1.20 (95%CI 0.81-1.79)  Recurrent fallers (≥3)  I: 43, C: 44, OR: 0.97 (95%CI 0.60-1.56) | Injurious fallers  I: 34 (17%), C 35 (18%)  OR: 0.97 (95% CI 0.57–1.62)  Time to first injurious fall  HR: 0.93 (95%CI 0.58–1.49)  Hip fractures:  I: 6 (3%), C: 11 (6%) | Nr of fall related ED revisits:  I: 41, C: 31 | Nr of fall related hospital admission:  I: 39 (20%) C: 16 (8%) | Death during FU: 4 (2%) in both groups  QoL SF-36 physical: I: 67.9 (25), C: 65.2 (27), p 0.04  SF-36 mental: I: 81.5 (18), C: 78.1 (23), p 0.39  SF General health: I: 70.0 (19), C: 66.1 (24.1), p 0.49 | Barthel score (SD)  I: 97.1 (9.3) C: 98.3 (3.6), p 0.10  FAI score (SD)  I: 30.1 (6.9) C: 29.4 (7.3), p 0.71  ABC score (SD)  I: 77.9 (18), C: 77.9 (21), p 0.77 |
| de Vries 2010 (19) | Nr of falls, median (IQR)  I: 1 (0-3), C: 1 (0-2)  Time to first fall  HR: 0.96 (95% CI 0.67-1.37) | Fallers:  I: 55 (52%), C: 62 (56%)  Recurrent fallers (≥2)  I: 37 (35%), C: 35 (32%) | Fractures  I: 5, C: 5, HR, 0.99 (95% CI, 0.73-1.34) | - | - | Death during FU  I: 1, C: 7  EuroQol mean Δ (SD), I: 0.01 (0.16), C: 0.07 (0.16), RC −0.17 (95%CI −1.31-0.97)  SF-12 mental mean Δ (SD):  Women: I: -1.3 (11.4), C: −1.0 (10.5), RC 0.6 (95%CI −3.7-2.6)  Men: I: 1.8 (11.1), C: −2.6 (9.6), RC 4.4 (−7.7-16.4)  SF-12 Physical mean Δ (SD): I: 2.6 (8.6), C: 1.9 (8.8), RC 0.7 (95%CI -2.2 - 3.5) | Barthel Index, mean Δ  I: −0.2 (SD 2.2), C: 0.2 (SD 1.9), RC −0.2 (−1.3 - 0.97)  Lawton iADL, mean Δ  Women:  I: 0.1 (SD 1,5), C: -0,1 (SD 1.5), RC 0.2 (95%CI –0.9 - 1.3)  Men:  I: –0.7 (SD 2.0), C: 0.3 (SD 1.9), RC –1 (95%CI -3.6 - 1.7)  Physical performance:  I: -1.1 (3.1), C: -0.7 (3.4), RC −0.4 (95%CI -2-1.3) |
| Whitehead 2003 (20) | - | Fallers  I: 28 C: 15. aOR 1.7 (95%CI 0.7-4.4) p 0.244 | - | - | - | - | - |
| Non-Randomised Controlled Trials | | | | | | | |
| Bogucki 2023 (21) | - | - | - | AC ED revisit  30 days: I: 259 (18%), C: 447 (29%); p <0.001  60 days: I: 390 (28%), C: 545 (40%) p <0.001;  90 days: I: 491 (35%); C: 576 (46%) p <0.001 | - | - | - |
| Clementz 2019 (22) | - | - | - | - | - | Death during FU  90 days: I: 11 (9%) C: 19 (15%) | - |
| Fulbrook 2025 (23) |  |  |  | Fall related ED revisits  30 days: I: 9 (3%) C: 15 (5%) p 0.215  90 days: I: 22 (7%) C: 23 (7%) p 0.877  AC ED revisits  30 days: I: 46 (14%) C: 37 (12%) p 0.410  90 days: I: 79 (25%) C: 83 (26%) p 0.927 |  |  |  |
| Harper 2013 (24) |  |  |  | Fall related ED revisits  I: 106 (6%), C: 68 (5%).  iRR: 1.29 (95%CI 0.95-1.78), p 0.09 | AC hospital readmissions  I: 77 (4%), C: 46 (3%);  iRR 1.14 (0.79-1.64), p 0.45 |  |  |
| Leahy 2014 (25) | - | - | - | Fall related ED revisits: I: 8 (17%), non-completion group: 9 (36%), non-referral group: 20 (47%); p 0.009 | - | - | - |
| Hepkema 2022 (26) | - | Fallers  I: 3 (20%), C 6 (32%) | - | - | - | - | - |
| Pre-Post Intervention Studies | | | | | | | |
| Ageron 2016 (27) | - | - | - | Fall related ED revisits  30 days: I: 17 (2%), C: 29 (4%), p 0.05. aOR: 0.52 (95CI% 0.28-0.95) p 0.035 | - | - | - |
| Baraff 1999 (28) | Nr of falls  I: 216, C: 328  Falls/100 personyears  I: 36.2 C: 36.2, p 0.993 | Fallers  I: 126 (21%), C: 165 (18%)  Recurrent fallers (≥2)  I: 48 (8%); C: 88 (10%) | Injurious fall with hospitalization  I: 24 (3%), C: 33 (3%)  Hip fractures  I: 10 (1%), C 12 (1%) | - | Fall related hospital admissions  I: 27 (4%), C: 43 (4%) | - | Regular exercise  I: 60%, C: 56% |
| Rosario 2023 (29) | - | - | - | AC ED revisit  7 days I: 1 (1%) C: 5 (4%) p 0.231  30 days I: 10 (10%), C: 14 (12%) p 0.831 | AC hospital admission  30 days I: 6 (6%) C: 11 (9%) p 0.612  6 months I: 29 (30%) C: 32 (27%) p 0.547 | Death during FU  I: 2 (2%) C: 5 (4%) p 0.468 | - |
| Jusmanova 2021 (30) Ω | - | - | - | - | AC hospital admission  I: 92 (13%) C: 124 (17%); p 0.032 | - | - |

Abbreviations: AC: AC; ADL: Activities of Daily Living; C: control group; FAI: Franchay activity index; FR: Fall related; I: intervention group; IRR: incidence rate ratio; ISS: Injury Severity Score, OR: Odds Ratio, Part.: participants; RR: relative risk, FIM/FAM: Functional independence and assessment measure; QoL: quality of life; Δ: difference. Unless otherwise specified, numbers reflect mean values and the standard deviation (SD). Ω: This study had no specific age requirement for inclusion; from this study results were obtained specifically of those patients aged 60 years and over to allow inclusion of their data by the original authors.

**References**

1. Barker A, Cameron P, Flicker L, Arendts G, Brand C, Etherton-Beer C, et al. Evaluation of RESPOND, a patient-centred program to prevent falls in older people presenting to the emergency department with a fall: A randomised controlled trial. PLoS medicine. 2019;16(5):e1002807.

2. Benhamou J, Espejo T, Riedel HB, Dreher-Hummel T, García-Martínez A, Gubler-Gut B, et al. On-site physiotherapy in older emergency department patients following a fall: a randomized controlled trial. European Geriatric Medicine. 2025;16(1):205–17.

3. Boyé ND, Van der Velde N, De Vries OJ, Van Lieshout EM, Hartholt KA, Mattace-Raso FU, et al. Effectiveness of medication withdrawal in older fallers: results from the Improving Medication Prescribing to reduce Risk Of FALLs (IMPROveFALL) trial. Age and ageing. 2017;46(1):142–6.

4. Polinder S, Boyé ND, Mattace-Raso FU, Van der Velde N, Hartholt KA, De Vries OJ, et al. Cost-utility of medication withdrawal in older fallers: results from the improving medication prescribing to reduce risk of FALLs (IMPROveFALL) trial. BMC geriatrics. 2016;16(1):179.

5. Chu MML, Fong KNK, Lit ACH, Rainer TH, Cheng SWC, Au FLY, et al. An occupational therapy fall reduction home visit program for community‐dwelling older adults in Hong Kong after an emergency department visit for a fall. Journal of the American Geriatrics Society. 2017;65(2):364–72.

6. Close J, Ellis M, Hooper R, Glucksman E, Jackson S, Swift C. Prevention of falls in the elderly trial (PROFET): a randomised controlled trial. The Lancet. 1999;353(9147):93–7.

7. Dadgari A, Rahmani P, Mirrezaie SM. The effect of nursing discharge planning program to prevent recurrent falls, readmission, and length of hospital stay in the aged patients: a randomized controlled trial. Topics in Geriatric Rehabilitation. 2022;38(4):277–84.

8. Davison J, Bond J, Dawson P, Steen IN, Kenny RA. Patients with recurrent falls attending Accident & Emergency benefit from multifactorial intervention—a randomised controlled trial. Age and ageing. 2005;34(2):162–8.

9. Goldberg EM, Marks SJ, Resnik LJ, Long S, Mellott H, Merchant RC. Can an emergency department–initiated intervention prevent subsequent falls and health care use in older adults? A randomized controlled trial. Annals of emergency medicine. 2020;76(6):739–50.

10. Harper KJ, Barton AD, Bharat C, Petta AC, Edwards DG, Arendts G, et al. Risk assessment and the impact of point of contact intervention following emergency department presentation with a fall. Physical & Occupational Therapy In Geriatrics. 2017;35(3-4):182–94.

11. Hendriks MRC, Bleijlevens MHC, Van Haastregt JCM, Crebolder HFJM, Diederiks JPM, Evers SMAA, et al. Lack of Effectiveness of a Multidisciplinary Fall-Prevention Program in Elderly People at Risk: A Randomized, Controlled Trial. Journal of the American Geriatrics Society. 2008;56(8):1390–7.

12. Lightbody E, Watkins C, Leathley M, Sharma A, Lye M. Evaluation of a nurse‐led falls prevention programme versus usual care: a randomized controlled trial. Age and ageing. 2002;31(3):203–10.

13. Matchar DB, Duncan PW, Lien CT, Ong MEH, Lee M, Gao F, et al. Randomized controlled trial of screening, risk modification, and physical therapy to prevent falls among the elderly recently discharged from the emergency department to the community: the steps to avoid falls in the elderly study. Archives of physical medicine and rehabilitation. 2017;98(6):1086–96.

14. Matchar DB, Eom K, Duncan PW, Lee M, Sim R, Sivapragasam NR, et al. A cost-effectiveness analysis of a randomized control trial of a tailored, multifactorial program to prevent falls among the community-dwelling elderly. Archives of physical medicine and rehabilitation. 2019;100(1):1–8.

15. Russell MA, Hill KD, Day LM, Blackberry I, Schwartz J, Giummarra MJ, et al. A randomized controlled trial of a multifactorial falls prevention intervention for older fallers presenting to emergency departments. Journal of the American Geriatrics Society. 2010;58(12):2265–74.

16. Shaw FE, Bond J, Richardson DA, Dawson P, Steen IN, McKeith IG, et al. Multifactorial intervention after a fall in older people with cognitive impairment and dementia presenting to the accident and emergency department: randomised controlled trial. Bmj. 2003;326(7380):73.

17. Vind AB, Andersen HE, Pedersen KD, Jørgensen T, Schwarz P. An outpatient multifactorial falls prevention intervention does not reduce falls in high‐risk elderly Danes. Journal of the American Geriatrics Society. 2009;57(6):971–7.

18. Vind AB, Andersen HE, Pedersen KD, Joergensen T, Schwarz P. Effect of a program of multifactorial fall prevention on health-related quality of life, functional ability, fear of falling and psychological well-being. A randomized controlled trial. Aging clinical and experimental research. 2010;22(3):249–54.

19. De Vries OJ, Elders PJ, Muller M, Knol DL, Danner SA, Bouter LM, et al. Multifactorial intervention to reduce falls in older people at high risk of recurrent falls: a randomized controlled trial. Archives of internal medicine. 2010;170(13):1110–7.

20. Whitehead C, Wundke R, Crotty M, Finucane P. Evidence-based clinical practice in falls prevention: a randomised controlled trial of a falls prevention service. Australian Health Review. 2003;26(3):88–97.

21. Bogucki S, Siddiqui G, Carter R, McGovern J, Dziura J, Gan G, et al. Effect of a home health and safety intervention on emergency department use in the frail elderly: a prospective observational study. Western Journal of Emergency Medicine. 2023;24(3):522.

22. Clementz A, Jost J, Lacour A, Bimou C, Gayot C, Ratsimbazafy V, et al. Effect of clinical pharmacy services in an older adult emergency medicine unit on unplanned rehospitalization of older adults admitted for falls: MUPA-PHARM study. Journal of the American Medical Directors Association. 2019;20(8):947–8.

23. Fulbrook P, Miles SJ, Jordan F, Hazelwood S, Lee HYD. Screening and assessment of falls risk in the emergency department. Australas Emerg Care. 2025;28(3):179–85.

24. Harper KJ, Gibson NP, Barton AD, Petta AC, Pearson SK, Celenza A. Effects of emergency department Care Coordination Team referrals in older people presenting with a fall. Emergency Medicine Australasia. 2013;25(4):324–33.

25. Leahy C, Chan D. Day hospital fall prevention programme for elderly people to reduce re-presentation with fall. Asian journal of gerontology and geriatrics. 2014;9(2):67–70.

26. Hepkema BW, Köster L, Geleijn E, Van den Ende E, Tahir L, Osté J, et al. Feasibility of a new multifactorial fall prevention assessment and personalized intervention among older people recently discharged from the emergency department. PLoS One. 2022;17(6):e0268682.

27. Ageron FX, Ricard C, Perrin‐Besson S, Picot F, Dumont O, Cabillic S, et al. Effectiveness of a multimodal intervention program for older individuals presenting to the emergency department after a fall in the northern French Alps emergency network. Academic emergency medicine. 2016;23(9):1031–9.

28. Baraff LJ, Lee TJ, Kader S, Penna RD. Effect of a practice guideline for emergency department care of falls in elder patients on subsequent falls and hospitalizations for injuries. Academic emergency medicine. 1999;6(12):1224–31.

29. Rosario BH, Yi-En CS, Barrera VC, Diraviyam B, Low SL, Lien C, et al. Emergency department falls interventions improve osteoporosis management in frail older adults. Annals of the Academy of Medicine, Singapore. 2023;52(6):327–30.

30. Jusmanova K, Rice C, Bourke R, Lavan A, McMahon CG, Cunningham C, et al. Impact of a specialist service in the emergency department on admission, length of stay and readmission of patients presenting with falls, syncope and dizziness. QJM: An International Journal of Medicine. 2021;114(1):32–8.
